# Effects of colon-targeted vitamins on the composition and metabolic activity of the human gut microbiome– a pilot study

**DOI:** 10.1080/19490976.2021.1875774

**Published:** 2021-02-21

**Authors:** Van T. Pham, Sophie Fehlbaum, Nicole Seifert, Nathalie Richard, Maaike J. Bruins, Wilbert Sybesma, Ateequr Rehman, Robert E. Steinert

**Affiliations:** a R&D Human Nutrition and Health, DSM Nutritional Products Ltd., Basel, Switzerland; b Department of Surgery, Division of Visceral and Transplantation Surgery, University Hospital Zurich, Zurich, Switzerland

**Keywords:** Vitamins, gut microbiome, dysbiosis, targeted delivery

## Abstract

An increasing body of evidence has shown that gut microbiota imbalances are linked to diseases. Currently, the possibility of regulating gut microbiota to reverse these perturbations by developing novel therapeutic and preventive strategies is being extensively investigated. The modulatory effect of vitamins on the gut microbiome and related host health benefits remain largely unclear. We investigated the effects of colon-delivered vitamins A, B2, C, D, and E on the gut microbiota using a human clinical study and batch fermentation experiments, in combination with cell models for the assessment of barrier and immune functions. Vitamins C, B2, and D may modulate the human gut microbiome in terms of metabolic activity and bacterial composition. The most distinct effect was that of vitamin C, which significantly increased microbial alpha diversity and fecal short-chain fatty acids compared to the placebo. The remaining vitamins tested showed similar effects on microbial diversity, composition, and/or metabolic activity *in vitro*, but in varying degrees. Here, we showed that vitamins may modulate the human gut microbiome. Follow-up studies investigating targeted delivery of vitamins to the colon may help clarify the clinical significance of this novel concept for treating and preventing dysbiotic microbiota-related human diseases. Trial registration: ClinicalTrials.gov, NCT03668964. Registered 13 September 2018 – Retrospectively registered, https://clinicaltrials.gov/ct2/show/NCT03668964.

## Background

Evidence increasingly indicates that imbalances in the human gut microbiota (HGM) – dysbiosis – may be associated with Western diseases, including obesity and type 2 diabetes, as well as cardiovascular, autoimmune, and intestinal inflammatory disease.^1^ Thus, targeted modulation of the HGM intended to restore imbalances represents a potential therapeutic and preventive strategy and has attracted the attention of academics as well as those engaged in various industries. Public awareness and acceptance of substances that modulate the HGM continue to grow. Although there is a general consensus regarding the beneficial effects of prebiotics and probiotics, there is still a lack of understanding of the exact benefits these provide at an individual level, their precise modes of action, and whether ingredients other than the traditional pre- and probiotics may beneficially modulate the HGM. The term “prebiotic” was originally defined as “a nondigestible food ingredient that beneficially affects the host by selectively stimulating the growth and/or activity of one or a limited number of bacteria in the colon, and thus improve host health” .^2(p1405)^ Over the past decades, this definition has been refined, and a more nuanced interpretation which expanded prebiotics beyond originally researched ingredients – such as fructooligosaccharides (FOS), galactooligosaccharides (GOS) and inulin – emerged. As a result, many novel prebiotic candidates including xylooligosaccharides (XOS), mannan oligosaccharide (MOS), and fermentable fibers such as beta-glucans, polyunsaturated fatty acids, and phenolic compounds have been recognized. The most recent definition by the International Scientific Association for Probiotics and Prebiotics (ISAPP) in 2016 also includes substrates, such as vitamins, that may affect microbiota composition via mechanisms not involving selective utilization by host microorganisms.^3^ Recent intervention studies that used high vitamin doses or colon-targeting formulations have shown that vitamins can impact gut microbiota. A 14 d supplement of 100 mg riboflavin increased the number of butyrate producers, namely *Faecalibacterium prausnitzii* and *Roseburia*, per gram of feces in healthy subjects,^4^ and decreased *Enterobacteriaceae* in patients with inflammatory bowel disease (IBD).^5^ Moreover, microencapsulated delayed-release niacin significantly increased the relative abundance of *Bacteroidetes*, and this increase was associated with improved biomarkers for systemic insulin sensitivity and metabolic inflammation.^6^ The current study further investigated the effect of selected vitamins, namely vitamin B2 (riboflavin), vitamin C (ascorbic acid), vitamin E (alpha-tocopherol), vitamin D (cholecalciferol-D3), vitamin A (retinol), and folic acid on gut microbial composition and related metabolic activity in human subjects by colon-targeted delivery of vitamins. In addition, a set of *in vitro* experiments including a short-term batch fermentation combined with cell models were performed to study the effects of these vitamins on barrier function and immune response.

## Methods

### Clinical study

#### Ethics statement

The study protocol was approved by the Clinical Research Ethics Committee of the Cork Teaching Hospitals (Cork, Ireland) (Protocol Number: AFCRO-087) and performed in accordance with the Declaration of Helsinki. Each subject provided written informed consent prior to inclusion in the study. The trial was registered with clinicaltrials.gov under the ID: NCT03668964.

#### Subjects

A total of 96 healthy volunteers, 12 in each of 6 distinct vitamin groups (vitamin A, vitamin B2, vitamin C, vitamin B2 + C, vitamin D3, and vitamin E) and 24 in the placebo group were involved in the study (Table S1). The main exclusion criteria were as follows: significant acute or chronic disease; smoking; pregnancy; antibiotic use within the previous 3 months; a history of drug and/or alcohol abuse (more than 2 servings/day); major dietary changes in the past 3 months; eating disorders; vegetarians or vegans; enemas; dietary supplements including prebiotic, probiotic, or fiber-rich supplements within 4 weeks prior to baseline visit, and for the duration of the intervention; high fiber diets (i.e. >30 g); chronic medications for active gastrointestinal disorders (unless the product was taken for at least 2 months prior to screening and the same dosage was maintained throughout the study); a recent change in bowel habits (<3 months); and abdominal pain.

All 96 participants completed the intervention, with no premature discontinuations. All participants were evaluated on a case by case basis by an independent committee before unblinding of the data, to determine inclusion to the per-protocol population. As a result, two subjects were excluded from the per-protocol fecal analysis. One participant in the vitamin A group took antibiotic due to a tonsillitis and one participant in the Vitamin E group was deemed non-compliant (compliance 75%).

#### Study Design

The trial was designed as a double-blind, randomized, placebo-controlled, parallel arm study in which subjects received either the vitamin supplement or placebo daily over a period of 4 weeks. There were three visits: (i) screening; (ii) baseline (1 week after screening); and (iii) follow-up (4 weeks after baseline). During screening, a Food Frequency Questionnaire (FFQ) was completed, vital signs were recorded, and a full medical examination – including medical history and a demographic and anthropometric assessment – was performed. In addition, a fasting venous blood sample was taken for safety profiling (full blood count, chemistry, glucose, and high-sensitivity C-reactive protein (hs-CRP)) and analysis of plasma vitamin concentration; the sample was stored at −80°C until analysis. Subjects completed two questionnaires: a 36-item Short Form Health survey questionnaire (SF-36), and the Gastrointestinal Symptom Rating Scale (GSRS). In addition, during the run-in phase (between the screening visit and the baseline visit), all volunteers were asked to record bowel movements and gastrointestinal symptoms utilizing GSRS, via a mobile phone application. At baseline, subjects were randomized to receive 1 of the 6 vitamin products or a placebo daily for 28 d. At follow-up visits, 4 weeks after baseline, the subjects received another medical examination, including blood sampling for safety parameters and vitamin level analysis. The subjects completed the SF-36 and GSRS questionnaires again.

A total of two stool samples were collected from each subject at baseline and after 4 weeks of intervention. The subjects were asked to collect a stool sample at home within 48 h before each visit, using a stool collection kit. The stool specimen was stored in the subject’s home freezer until the next visit, when it was stored at −20°C.

#### Investigational products

The products investigated were as follows: i) vitamin A (250 µg retinol equivalents (RE)/day); (ii) vitamin B2 (75 mg riboflavin/day); (iii) vitamin C (500 mg ascorbic acid/day); (iv) vitamin B2 (75 mg/day) + vitamin C (500 mg/day); (v) vitamin D3 (60 µg cholecalciferol/day); (vi) vitamin E (100 alpha-tocopherol equivalents mg/day); and a placebo (200 mg/day microcrystalline cellulose). All vitamins were provided by DSM Nutritional Products Ltd. (Kaiseraugst, Switzerland). The placebo was obtained from Fagron (Waregem, Belgium). Each investigational product was formulated as a colon-release form in a hard gelatin capsule (Lonza, Bornem, Belgium) coated using a pH-dependent polymer, Eudragit S100 (Evonik Nutrition & Care GmbH, Darmstadt, Germany), which had been validated for targeted colon delivery.^7^ The selection of dosage was based on subtracting estimated intestinal absorption levels for each vitamin^8–11^ from high oral doses of vitamins used in previous studies .^4,12–15^ All doses were below the upper limits published by the European Food Safety Authority (EFSA), except vitamin B2, for which no upper limit has been established.^16^

#### Measurements

***Fecal microbial composition***. Total DNA was extracted from all collected fecal samples using a QIAamp DNA Stool Minikit (Qiagen, Crawley, United Kingdom) according to the manufacturer’s instructions, apart from the addition of a bead-beating step and increasing the lysis temperature to 95°C as described previously.^17^ Isolated DNA was quantified using a Qubit High-Sensitivity DNA assay (Thermo Fisher, Waltham, MA USA). Whole metagenome libraries were then prepared using an Illumina Nextera XT kit (Illumina, San Diego, USA) in accordance with the manufacturer’s instructions, with the following modifications: tagmentation time was increased to 7 min, and the samples were each individually sized by running on an Agilent High-Sensitivity Chip (Agilent) and quantified using the Qubit High-Sensitivity DNA assay (Thermo Fisher, Waltham, MA USA) in accordance with Teagasc Sequencing Platform SOPs, following the incorporation of indices and Ampure purification of products. Next, the samples were pooled equimolarly and sequenced on an Illumina NextSeq 500 with a NextSeq 500/550 v2 high-output reagent kit (300 cycles). All sequencing was conducted at the Teagasc sequencing facility in accordance with standard Illumina sequencing protocols. Delivered raw FASTQ sequence files were quality checked for poor quality and duplicate read removal, and trimmed via a combination of SAM and Picard tools. Taxonomic assignment of the reads was completed using MetaPhlAn 2.0 software and functional analysis was performed with SUPER-FOCUS. Alpha and beta diversity analyses were performed using R (R Core Team, Vienna, Austria).^18,19^

***Fecal short-chain fatty acids (SCFA).*** Fecal SCFA were extracted and measured via gas chromatography-mass spectrometry (GC-MS) by MS-Omics (Copenhagen, Denmark), based on a previously established method.^20^

***Fecal ammonia***. Fecal ammonia levels were determined using an Ammonia Assay Kit (ab83360, Abcam). Fecal pellets were suspended in the provided assay buffer at a concentration of 1 mg/10 µl and centrifuged at 13,000 x *g* for 10 min at room temperature (°C) to remove insoluble material. Ammonia concentrations were then determined according to the kit protocol.

***Plasma and fecal vitamin B2 concentration***. Plasma and fecal vitamin B2 concentrations were measured via liquid chromatography. Detailed methods are provided in Supplementary file 1.

***Fecal redox and pH balance.*** A pH and redox meter (PCE-228-R, PCE Instruments, Southampton, United Kingdom) was used to measure the pH and the redox potential as described previously.^21^

***Quality of life and gastrointestinal symptoms questionnaires.*** The Short-Form Health Survey (SF-36)^22^ was used to assess the quality of life. The 36 items in SF-36 covered 8 domains: physical functioning, limitations due to physical health, bodily pain, general health, vitality, social functioning, and limitations related to emotional and mental health. The results were evaluated by attributing scores to each question, which were then transformed into a scale ranging from 0 to 100, with a higher score indicating better health status.

Gastrointestinal symptoms were assessed using the Gastrointestinal Symptom Rating Scale (GSRS).^23^ GSRS utilizes a 7-point rating scale, depending on the intensity and frequency of GI symptoms experienced during the previous weeks. A higher score indicates more inconvenient symptoms.

### In vitro fermentation study

#### Donors and sample preparation

Three fecal donors (male, 26 years; female, 35 years; female, 29 years) were pre-screened in a short-term colonic fermentation experiment to select one donor with a balanced SCFA production profile to be included in the final fermentation experiment. All donors were healthy, free from any known gastrointestinal disease, on a normal diet (i.e. no major dietary changes in the past 3 months; no eating disorders; no vegetarians or vegans; no additional vitamin supplements), and without any history of antibiotic use during the previous 6 months. Fecal material was collected from each donor to prepare slurries in an anaerobic phosphate buffer. A 10% (v/v) concentration of these fecal slurries was added to sugar-depleted nutritional colon medium mimicking colon basal nutrients and supplemented with NaCl, MgSO_4_⋅7H_2_O, CaCl_2_⋅2H_2_O, hemin, and bile salts. Glucose, starch, and cellobiose were added as a carbon source. Each incubation was performed for 48 h at 37°C while shaking (90 rpm) under anaerobic conditions, and repeated once. Samples were collected after 48 h.

For the final batch fermentation experiment, all test ingredients from stock solutions were added to a modified nutritional medium, containing (g/l): 2.5 K_2_HPO_4_, 10.9 KH_2_PO_4_, 2 NaHCO_3_, 2 yeast extract, 2 peptone, 1 mucin, 0.5 cysteine, 2 Tween 80, 2 glucose, 2 starch, 2 cellobiose, 0.1 NaCl, 0.01 MgSO_4_⋅7H_2_O, 0.01 CaCl_2_⋅6H_2_O, 0.05 hemin, and 0.5 bile salts. Vitamins were added from stock solutions prepared in water and tested in three different concentrations: 0.2x, 1x, and 5x. A fresh fecal suspension prepared from the feces of each selected donor, representing the microbial source, was added to the reactors. Each reactor had a volume of 70 mL. All tests except blanks were repeated once, resulting in 24 independent incubations **(Table S2)**. Incubation was performed for 48 h at 37°C while shaking (90 rpm) under anaerobic conditions. Effluent samples were collected from each fermentation flask before (0 h fermentation) and after fermentation (48 h fermentation), sterilized by filtering through 0.22 µm filters. Samples were used to analyze microbiome composition and SCFA as well as to conduct *in vitro* analysis in cell culture systems (immune and barrier function).

#### Measurements

***Fecal microbial composition.*** DNA extraction and sequencing were performed via the same method used for the clinical trial of fecal samples.

***In vitro microbial metabolic activity.*** pH (Senseline F410; ProSense, Oosterhout, The Netherlands), gas pressure (Hand-held pressure indicator CPH6200; Wika, Echt, The Netherlands), and SCFA (acetate, propionate, and butyrate) were measured at the start of incubation, after 24 and after 48 h. SCFA were extracted and measured by gas chromatography as described previously.^24^

***Caco-2 and HT29-MTX-E12 cell culture and barrier function.*** CaCo-2 (ECACC 86010202) and the mucus-secreting HT29-MTX-E12 cells (ECACC 12040401) were both purchased from the European Collection of Authenticated Cell Cultures (Salisbury, UK). The two cell lines were cultured separately in Falcon tissue culture ﬂasks (Corning Life Sciences B.V., Amsterdam, Netherlands) in a complete growth medium as described previously.^25^ To assess the effect of batch fermentation supernatants on intestinal barrier integrity, co-cultures of Caco-2 and HT29-MTX-E12 cells were seeded at a density of 20,000 cells/well at a 7:3 ratio (Caco-2:HT29-MTX-E12) on the apical surface of Corning HTS Transwell-24 system PET membranes with a 0.4 µM pore size and a cell growth area of 0.33 cm^2^/well, and cultured in a complete growth medium that was renewed every second to third day. As previously described,^25^ the integrity of the monolayers was confirmed on day 14 post-seeding by measuring transepithelial electrical resistance (TEER) prior to apical treatment with 150 µl sterile filtered fermentation supernatants diluted 1:10 in complete growth medium. Experiments were conducted in technical triplicates per treatment and time point. Following a 48 h incubation period at 37°C in an atmosphere of 5% CO_2_, resistance across each cell monolayer was measured and the percentage change in TEER compared to initial TEER was calculated for each insert after subtracting the resistance value of the ﬁlters alone.

***HT29 cell culture and immune function.*** HT29 cells (ATCC® HTB-38™) were obtained from American Type Culture Collection (Manassas, VA, USA). HT29 cells were cultured and treated as previously described.^25^ Consequently, cytokines, chemokines, and interleukins in HT-29 supernatants, including GROa-CXCL1, IL8-CXCL8, and MIP3a-CCL20, were quantified using Luminex Technology (LiquiChip Workstation IS 200, Qiagen, Hilden, Germany) with Bio-Plex Pro Human Cytokine Panel kits (Bio-Rad, Hercules, CA) or Luminex Screening Assay kits (R&D Systems, Inc., Minneapolis, MN), following the manufacturer’s instructions. Data were acquired with Luminex IS 2.3 software and evaluated using LiquiChip Analyzer software (Qiagen, Hilden, Germany).

### Statistical analysis

Metagenomic sequencing data, including taxonomy and alpha diversity, were analyzed using non-parametric tests due to deviation from normality. Within-group changes before and after colon-delivered vitamin intervention were assessed using Wilcoxon signed-rank tests for paired data, while changes between groups were analyzed using Wilcoxon rank sum tests. Beta diversity was analyzed using a Bray–Curtis distance matrix and significance was calculated using Adonis.

The statistical analyses of all other clinical outcomes were performed using the following methodology. Parametric assumptions were evaluated and the differences between parametric data were assessed using Analysis of Variance (ANOVA). A mixed 2 × 7 ANOVA was used to compare the data at baseline and follow-up, of which the comparisons between each vitamin group versus placebo were reported. In addition, one-way ANOVAs or unpaired t-tests (as appropriate) were used to determine significant differences between absolute changes from baseline to follow-up, estimated for each vitamin group and the placebo. For data that did not meet parametric assumptions, non-parametric approaches were adopted. Pairwise post-hoc comparisons were performed. All values are expressed as means (± SEM) and statistical significance was set at *p* < .05. All analyses are based on the per-protocol population.

*In vitro* data on immune function and gut barrier integrity were analyzed using linear models, including categorical dose, time, and interaction as fixed effects. Within each model, data from all doses of the respective vitamin were entered as well as the control group data, which represented a dose of 0.

R (R Core Team, Vienna, Austria) and SPSS 22 (IBM Corp, Armonk NY, USA) were used for analyses.^26^

## Results

### Clinical study

#### Colon-delivered vitamins modulate gut microbial composition

**Figure 1.**
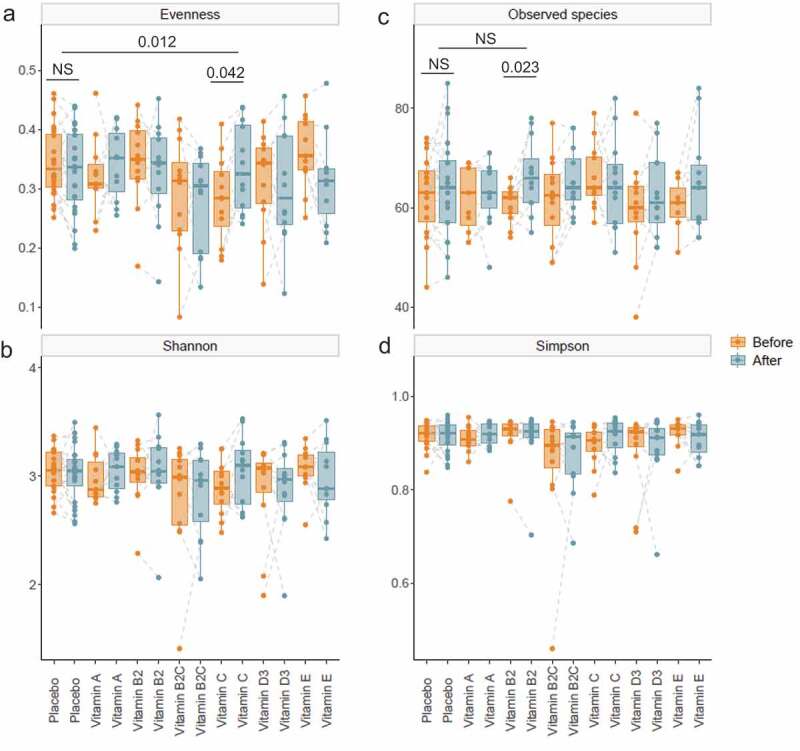
**Alpha diversity of gut microbiota before and after colon-delivered vitamin intervention.**Diversity indices, including evenness (a), Shannon’s index (b), observed number of species (c) and Simpson’s index (d) were compared before and after colon-delivered vitamin intervention, using a paired Wilcoxon test. Absolute changes between the intervention group and the placebo were compared using a Wilcoxon test. Values are shown as median and interquartile range. NS, not significant, *p* > .05

We compared different alpha diversity indices within groups as well as between treatment and placebo groups (Figure 1). The results indicated that vitamin C significantly increased gut microbial evenness at week four when compared with that of the baseline (*p* = .042) and placebo (*p* = .012) groups. Moreover, vitamin B2 significantly increased the observed number of species compared to the baseline (*p* = .023). Permutational multivariate analysis of variance (PERMANOVA) of Bray–Curtis distances suggested that the overall changes in gut microbial beta diversity were not significantly different between groups or when compared to baseline within groups **(Table S3)**.

**Figure 2.**
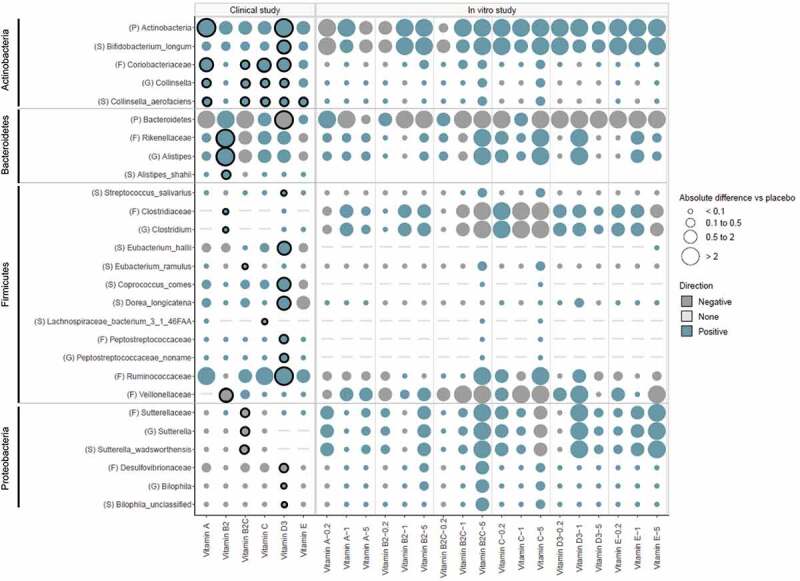
**Effect of vitamin treatments on microbial composition in humans and *in vitro***. Values are shown as absolute difference in relative abundance at the phylum (p), family (f), genus (g), and species (s) level versus placebo (for human study), or versus the control (for *in vitro* study), using different bubble size. Direction of change is depicted by color. Significant differences are marked as bold

Various vitamins caused changes in gut microbial composition within groups (Table 1) as well as between treatment and placebo groups (Figure 2) at the phylum, family, genus and species level.

**Table 1.** Changes in microbial composition in response to colon-delivered vitamins within groups before and after intervention

**Microbial taxa^1^**	**Placebo**			**Vitamin A**			**Vitamin B2**		
	**Before**	**After**	**Fold change**	**Before**	**After**	**Fold change**	**Before**	**After**	**Fold change**
**Phylum**									
*Actinobacteria*	7.1[3.79,14.00]**^2^**	5.33 [2.30,10.61]	1.33 ↓P=0.152	3.28 [1.77,6.64]	3.57 [2.78,9.21]	1.09 ↑ P=0.175	2.57[1.42,6.88]	2.34[1.18,4.40]	1.10 ↓P=0.569
*Bacteroidetes*	34.29 [15.8,53.24]	35.37 [24.6,45.67]	1.03 ↑ P=0.900	38.82 [25.10,58.05]	40.16 [25.9,52.22]	1.03 ↑ P=0.413	39.04 [14.77,52.23]	45.37 [34.5,61.82]	1.16 ↑ P=0.092
*Firmicutes*	55.43 [38.5,66.99]	56.33 [47.4,62.82]	1.02 ↑ P=0.989	44.96 [36.94,69.92]	47.3 [43.19,59.79]	1.05 ↑ P=0.765	54.41 [43.17,70.63]	42.56 [27.29,58.33]	1.28 ↓P=0.092
*Proteobacteria*	0.48 [0.21,1.04]	0.43 [0.27,0.83]	1.12 ↓P=0.790	0.64 [0.40,1.33]	0.55 [0.19,1.18]	1.16 ↓P=0.52	2.10[0.5,3.91]	0.77 [0.23,1.67]	2.73 ↓P=0.204
*Verrucomicrobia*	0.11[0.00,0.91]	0.21[0.00,1.57]	1.91 ↑ P=0.571	0.64 [0.35,0.91]	0.25 [0.02,1.91]	2.56 ↓P=0.541	0.06[0.00,0.61]	1.19 [0.37,2.66]	19.83 ↑ P=0.077
**Family**									
*Sutterellaceae*	0.15[0.00,0.27]	0.04[0.00,0.12]	3.75 ↓P=0.113	0.36 [0.27,0.42]	0.15 [0.05,0.44]	2.40 ↓P=0.577	0.15 [0.03,1.18]	0.05 [0.01,0.20]	3.00 ↓P=0.308
*Clostridiaceae*	0.03[0.00,0.15]	0.00[0.00,0.12]	NAP=0.603	0.00[0.00,1.16]	0.00[0.00,0.11]	NA**^4^**P=0.529	**0.00[0.00,0.02]**	**0.19 [0.01,2.46]**	NA* ↑ **P=0.009**
*Veillonellaceae*	0.19[0.00,2.04]	0.12[0.00,0.89]	1.58 ↓P=0.952	0.30 [0.05,2.09]	0.26[0.00,2.75]	1.15 ↓P=0.722	**2.18 [0.24,5.09]**	**1.20 [0.01,2.24]**	1.82* ↓**P=0.023**
*Lachnospiraceae*	13.42 [10.1,17.93]	15.88 [8.4,23.43]	1.18 ↑ P=0.229	**11.77 [9.96,16.93]**	**13.56 [11.21,21.23]**	1.15* ↑ **P=0.024**	16.74 [4.85,22.71]	7.30 [4.26,15.21]	2.29 ↓P=0.424
*Coriobacteriaceae*	1.85 [0.78,2.6]	1.33 [0.79,1.64]	0.72 ↓P=0.074	1.15 [0.48,1.38]	1.26 [1.05,2.03]	1.10 ↑ P=0.102	0.82 [0.55,1.22]	0.93 [0.43,1.29]	1.13 ↑ P=0.470
*Desulfovibrionaceae*	0.2 [0.04,0.29]	0.27 [0.07,0.43]	1.35 ↑ P=0.183	0.16 [0.10,0.34]	0.21 [0.07,0.25]	1.31 ↑ P=0.638	0.19[0.00,0.33]	0.21 [0.05,0.34]	1.11 ↑ P=0.554
**Genus**									
*Sutterella*	0.00[0.00,0.19]	0.00[0.00,0.09]	NAP=0.295	0.26 [0.02,0.36]	0.03[0.00,0.07]	8.67 ↓P=0.234	0.02[0.00,0.58]	0.02[0.00,0.08]	1.00 ↓P=0.108
*Clostridium*	0.03[0.00,0.15]	0[0.00,0.11]	0.00P=0.570	0.00[0.00,1.16]	0.00[0.00,0.11]	NAP=0.529	**0.00[0.00,0.02]**	**0.19[0.00,2.46]**	NA* ↑ **P=0.009**
*Faecalibacterium*	5.95 [4.08,8.64]	5.18 [2.51,10.69]	1.15 ↓P=0.944	6.03 [4.94,6.69]	6.69 [4.85,8.87]	1.11 ↑ P=0.520	**5.39 [3.35,7.58]**	**3.48 [1.63,4.74]**	1.55* ↓**P=0.012**
*Coprococcus*	2.20 [1.17,3.84]	2.03 [0.81,6.45]	1.08 ↓P=0.075	2.09 [0.85,9.82]	4.42 [1.27,11.31]	2.11 ↑ P=0.278	2.04 [1.48,5.48]	2.10 [0.59,3.59]	1.03 ↑ P=0.470
*Odoribacter*	0.02[0.00,1.83]	0.05[0.00,1.00]	2.50 ↑ P=0.449	0.9 [0.03,1.09]	0.09[0.00,0.68]	10.00P=0.080	1.07 [0.02,1.22]	1.20 [0.41,1.44]	1.12 ↑ P=0.519
**Species**									
*Sutterella wadsworthensis*	0.00[0.00,0.19]	0.00[0.00,0.09]	NAP=0.295	0.26 [0.02,0.36]	0.03[0.00,0.07]	8.67 ↓P=0.234	0.02[0.00,0.58]	0.02[0.00,0.08]	1.00P=0.108
*Streptococcus salivarius*	0.05 [0.02,0.21]	0.05[0.00,0.20]	1.00P=0.896	0.02[0.00,0.07]	0.05[0.00,0.08]	2.50 ↑ P=0.760	0.04[0.00,0.13]	0.05[0.00,0.10]	1.25 ↑ P=1.000
*Faecalibacterium prausnitzii*	5.95 [4.08,8.64]	5.18 [2.51,10.69]	1.15 ↓P=0.944	6.03 [4.94,6.69]	6.69 [4.85,8.87]	1.11 ↑ P=0.520	**5.39 [3.35,7.58]**	**3.48 [1.63,4.74]**	1.55* ↓P=0.012
*Dorea longicatena*	2.29 [0.87,4.34]	1.66 [0.46,4.51]	1.38 ↓P=0.768	2.84 [0.27,3.32]	2.36 [1.09,3.77]	1.20 ↓P=0.320	0.73 [0.49,1.64]	0.64 [0.37,1.87]	1.14 ↓P=0.677
*Coprococcus catus*	0.32 [0.09,0.45]	0.19 [0.05,0.29]	1.68 ↓P=0.095	0.24 [0.09,0.55]	0.22 [0.13,0.36]	1.09 ↓P=1.000	0.18 [0.13,0.25]	0.13 [0.07,0.23]	1.38 ↓P=0.129
*Alistipes senegalensis*	0.01[0.00,0.08]	0.02[0.00,0.06]	2.00 ↑ P=0.623	0.01[0.00,0.06]	0[0.00,0.08]	0.00 ↓P=0.834	0.01[0.00,0.12]	0.14 [0.01,0.23]	14.00 ↑ P=0.236
*Bifidobacterium longum*	1.50 [0.47,2.53]	0.89 [0.48,1.72]	1.69 ↓P=0.086	0.55 [0.24,1.95]	0.53 [0.36,1.37]	1.04 ↓P=1.000	0.63 [0.27,1.2]	0.40 [0.09,1.35]	1.58 ↓P=0.850
*Eubacterium hallii*	1.83 [0.79,3.53]	1.94 [0.95,3.42]	1.06 ↑ P=0.855	1.19 [0.54,2.58]	1.49 [0.82,2.43]	1.25 ↑ P=0.898	**1.16 [0.75,1.66]**	**0.71 [0.30,1.31]**	1.63* ↓**P=0.042**
*Coprococcus comes*	1.10 [0.42,1.88]	0.86 [0.27,1.3]	1.28 ↓P=0.224	0.70 [0.27,0.89]	0.78 [0.41,1.08]	1.11 ↑ P=0.413	0.75 [0.4,1.32]	0.54 [0.41,0.80]	1.39 ↓P=0.622
*Lachnospiraceae bacterium*	0.01 [0.00,0.15]	0.03[0.00,0.2]	3.00 ↑ P=0.344	0.12[0.00,0.57]	0.29 [0.03,0.45]	2.42 ↑ P=0.407	0.04 [0.01,0.14]	0.02[0.00,0.10]	2.00 ↓P=0.554
*Alistipes shahii*	0.44 [0.2,0.9]	0.43 [0.08,1.25]	1.02 ↓P=0.862	0.22 [0.05,0.96]	0.15 [0.04,0.89]	1.47 ↓P=0.813	**0.46 [0.12,0.87]**	**1.04 [0.51,1.97]**	2.26* ↑ **P=0.045**

**Microbial taxa^1^**	**Vitamin B2+C**			**Vitamin C**			**Vitamin D**			**Vitamin E**		
	**Before**	**After**	**Fold change**	**Before**	**After**	**Fold change**	**Before**	**After**	**Fold change**	**Before**	**After**	**Fold change**
**Phylum**												
*Actinobacteria*	1.69[1.37,4.51]	3.02[1.29,7.01]	1.79 ↑ P=0.910	2.60[1.30,9.28]	3.99[1.84,4.39]	1.53 ↑ P=0.910	2.71[1.89,11.17]	5.37[4.13,10.47]	1.98 ↑ P=0.20	1.97[1.08,5.52]	2.70[0.90,3.97]	1.37 ↑ P=0.966
*Bacteroidetes*	32.93 [28.92,56.82]	32.82 [18.62,44.9]	1.00P=0.064	46.44 [16.4,57.15]	44.89 [31.8,54.64]	1.03 ↓P=0.733	45.31[37.89,54.85]	23.27[14.79,45.36]	1.95 ↓P=0.052	49.67 [42.43,57.21]	45.19[29.35,60.27]	1.10 ↓P=0.966
*Firmicutes*	52.9 [38.12,64.12]	54.94 [48.03,70.7]	1.04 ↑ P=0.052	44.17 [36.5,64.48]	44.99 [34.4,57.95]	1.02 ↑ P=0.519	44.56[39.87,54.05]	58.19[46.31,70.35]	1.31 ↑ P=0.11	44.61 [35.61,51.28]	47.73[37.79,59.39]	1.07 ↑ P=0.831
*Proteobacteria*	**1.05 [0.79,1.54]**	**0.73 [0.23,1.20]**	1.44*P=0.021^3^	0.77 [0.42,1.78]	0.56 [0.41,2.38]	1.38 ↓P=0.910	0.98 [0.22,2.29]	0.42 [0.16,0.79]	2.33 ↓P=0.092	0.61 [0.38,0.89]	0.75 [0.66,1.26]	1.23 ↑ P=0.465
*Verrucomicrobia*	0.15[0.00,0.78]	0.02[0.00,0.66]	7.50 ↓P=0.554	0.02[0.00,0.23]	0.00[0.00,0.24]	0.00P=0.208	0.12[0.00,1.45]	0.12[0.00,4.32]	1.00P=0.141	1.25[0.00,2.82]	0.46[0.00,1.23]	2.72 ↓P=0.675
**Family**												
*Sutterellaceae*	**0.57 [0.16,0.95]**	**0.11[0.00,0.31]**	5.18*↓**P=0.005**	0.12 [0.06,0.76]	0.14 [0.05,0.31]	1.17 ↑ P=0.307	0.11[0.00,0.31]	0.02[0.00,0.13]	5.50 ↓P=0.080	0.06 [0.02,0.32]	0.02[0.00,0.29]	3.00 ↓P=0.906
*Clostridiaceae*	0.00[0.00,0.23]	0.07[0.00,0.22]	NA ↑ P=0.726	0.06[0,2.35]	0.02[0.00,3.91]	3.00 ↓P=0.813	0.00[0.00,0.05]	0.14 [0.01,0.62]	NA ↑ P=0.554	0.00[0.00,0.07]	0.05[0.00,0.16]	NA ↑ P=0.294
*Veillonellaceae*	1.25 [0.15,1.87]	2.3 [0.19,4.17]	1.84 ↑ P=0.126	0.21 [0.03,1.08]	0.61 [0.04,1.65]	2.90 ↑ P=0.185	0.04[0.00,3.17]	0.44 [0.01,8.02]	11.00 ↑P=0.636	0.05[0.00,2.19]	0.16 [0.03,1.19]	3.20 ↑ P=0.541
*Lachnospiraceae*	8.98 [6.73,10.95]	10.59 [6.25,17.37]	1.18 ↑ P=0.151	10.42 [7.85,18.01]	10.33 [6.52,12.41]	1.01 ↓P=0.424	9.09 [6.08,12.89]	13.66 [8.36,18.94]	1.50 ↑ P=0.151	12.95 [9.76,18.56]	9.91 [6.63,21.58]	1.31 ↓P=0.898
*Coriobacteriaceae*	0.56 [0.3,0.99]	0.65 [0.36,1.12]	1.16 ↑ P=0.266	0.54 [0.3,0.83]	1.15 [0.46,1.43]	2.13 ↑ P=0.266	**0.71 [0.41,0.98]**	**1.08 [0.91,1.46]**	1.52*↑**P=0.034**	0.59 [0.34,0.96]	0.64 [0.4,0.85]	1.08 ↑ P=0.638
*Desulfovibrionaceae*	0.19 [0.11,0.31]	0.15 [0.11,0.4]	1.27 ↓P=0.505	0.30 [0.11,0.35]	0.16 [0.03,0.31]	1.88 ↓P=0.625	**0.14 [0.04,0.23]**	**0.05 [0.00,0.08]**	2.80*↓**P=0.044**	0.18 [0.12,0.39]	0.20 [0.18,0.35]	1.11 ↑ P=1.000
**Genus**												
*Sutterella*	**0.48 [0.02,0.95]**	**0.03[0.00,0.25]**	16.00*↓**P=0.006**	0.08 [0.01,0.62]	0.07 [0.01,0.31]	1.14 ↓P=0.286	0.00[0.00,0.24]	0.00 [0.00,0.01]	NAP=0.178	0.00 [0.00,0.17]	0.00 [0.00,0.28]	NAP=0.834
*Clostridium*	0.00[0.00,0.23]	0.03[0.00,0.22]	NA ↑ P=0.726	0.05[0.00,2.35]	0.01[0.00,3.9]	5.00 ↓P=0.813	0.00[0.00,0.05]	0.14 [0.01,0.50]	NAP=0.554	0.00 [0.00,0.04]	0.04 [0.00,0.11]	NA ↑ P=0.294
*Faecalibacterium*	4.45 [3.19,7.93]	5.32[2.50,8.40]	1.20 ↑ P=0.677	4.66 [3.61,7.46]	5.63 [4.31,8.66]	1.21 ↓P=0.129	4.34 [3.73,5.85]	6.64 [2.14,10.19]	1.53 ↑ P=0.204	6.73 [4.43,9.68]	5.40 [3.89,6.22]	1.25 ↓P=0.067
*Coprococcus*	**1.26 [0.75,5.24]**	**1.53 [0.73,13.77]**	1.21* ↑ **P=0.027**	1.10 [0.51,5.09]	1.28 [0.44,2.75]	1.16 ↓P=0.563	1.73 [0.75,3.42]	2.06 [1.22,7.10]	1.19 ↑ P=0.519	5.52 [1.03,8.94]	4.82 [0.45,8.5]	1.15 ↓P=0.700
*Odoribacter*	0.91 [0.04,1.26]	0.21 [0.02,0.90]	4.33 ↓P=0.211	0.09[0.00,0.76]	0.57[0.00,1.41]	6.33 ↓P=1.000	**0.10[0.00,1.72]**	**0.00 [0.00,0.04]**	0.00*P=0.042	1.14 [0.06,1.94]	0.67 [0.05,2.31]	1.70 ↓P=0.554
**Species**												
*Sutterella wadsworthensis*	**0.48 [0.02,0.95]**	**0.03[0.00,0.25]**	16.00*↓**P=0.006**	0.08 [0.01,0.62]	0.07 [0.01,0.31]	1.14 ↓P=0.286	0.00[0.00,0.24]	0.00 [0.00,0.01]	NAP=0.178	0.00 [0.00,0.17]	0.00 [0.00,0.28]	NAP=0.834
*Streptococcus salivarius*	0.00[0.00,0.17]	0.04[0.00,0.13]	NA ↑ P=0.944	0.07 [0.01,0.17]	0.11 [0.03,0.31]	1.57 ↑ P=0.569	**0.00[0.00,0.02]**	**0.07 [0.03,0.17]**	NA* ↑ **P=0.009**	0.03 [0.00,0.07]	0.05 [0.01,0.08]	1.67 ↑ P=0.760
*Faecalibacterium prausnitzii*	4.45 [3.19,7.93]	5.32[2.50,8.40]	1.20 ↑ P=0.677	4.66 [3.61,7.46]	5.63 [4.31,8.66]	1.21 ↑ P=0.129	4.34 [3.73,5.85]	6.64 [2.14,10.19]	1.53 ↑ P=0.204	6.73 [4.43,9.68]	5.40 [3.89,6.22]	1.25 ↓P=0.067
*Dorea longicatena*	1.36 [0.57,1.84]	1.64 [0.78,2.35]	1.21 ↑ P=0.339	0.70 [0.53,1.25]	0.90 [0.56,2.29]	1.29 ↑ P=0.910	**1.04 [0.49,2.61]**	**1.97 [1.76,4.34]**	1.89* ↑**P=0.021**	1.36 [0.82,1.5]	0.43 [0.16,1.87]	3.16 ↓P=0.240
*Coprococcus catus*	**0.21 [0.14,0.32]**	**0.12 [0.04,0.20]**	1.75* ↓**P=0.027**	0.15 [0.05,0.26]	0.13 [0.01,0.16]	1.15 ↓P=0.398	0.28 [0.16,0.33]	0.27 [0.15,0.36]	1.04 ↓P=0.756	0.1 [0.06,0.24]	0.07 [0.03,0.45]	1.43 ↓P=0.838
*Alistipes senegalensis*	**0.05[0.00,0.10]**	**0.02[0.00,0.07]**	2.50* ↓**P=0.030**	0.01[0.00,0.02]	0.02[0.00,0.06]	2.00 ↑ P=0.236	0.00[0.00,0.03]	0.00 [0.00,0.02]	NAP=0.675	0.01 [0.00,0.09]	0.01 [0.00,0.08]	1.00P=0.722
*Bifidobacterium longum*	0.45 [0.06,1.64]	0.50 [0.11,1.39]	1.11 ↑ P=0.838	0.57 [0.25,1.26]	0.48 [0.14,1.2]	1.19 ↓P=0.850	**0.62 [0.22,1.25]**	**1.35 [0.81,2.33]**	2.18*↑ **P=0.037**	0.7 [0.06,1.71]	0.31 [0.09,1.1]	2.26 ↓P=0.760
*Eubacterium hallii*	0.97 [0.30,1.25]	0.81 [0.68,1.40]	1.20 ↓P=0.910	0.90 [0.35,1.87]	1.35 [0.86,2.7]	1.50 ↑ P=0.733	1.27 [0.37,3.17]	2.25 [1.37,4.29]	1.77 ↑ P=0.077	0.99 [0.70,1.88]	1.11 [0.37,2.2]	1.12 ↑ P=0.898
*Coprococcus comes*	0.50 [0.32,0.83]	0.68 [0.40,1.12]	1.36 ↑ P=0.110	0.53 [0.18,0.99]	0.54 [0.16,1.13]	1.02 ↑ P=0.760	**0.49 [0.26,0.56]**	**0.99 [0.34,1.16]**	2.02* ↑P=0.042	0.72 [0.36,0.82]	0.21 [0.04,0.43]	3.43 ↓P=0.415
*Lachnospiraceae bacterium*	0.01[0.00,0.04]	0.00[0.00,0.06]	0.00P=0.675	**0.04 [0.01,0.39]**	**0.00[0.00,0.19]**	0.00*P=0.044	0.04[0.00,0.35]	0.17 [0.00,0.24]	4.25 ↑ P=0.726	0.27 [0.00,0.43]	0.14 [0.01,0.37]	1.93 ↓P=0.813
*Alistipes shahii*	0.43 [0.25,0.68]	0.38 [0.16,0.54]	1.13 ↓P=0.351	0.44 [0.23,1.17]	0.80 [0.21,1.56]	1.82 ↑ P=0.351	0.22[0.00,0.65]	0.02 [0.00,0.80]	11.00 ↓P=1.000	0.72 [0.21,2.08]	0.84 [0.09,1.87]	1.17 ↑ P=0.683

1 All phyla and all other bacterial taxa showing a significant difference in at least one treatment group are shown.

2 Data are shown as median relative abundance [interquartile range].

3 * indicate significant difference with *P* < 0.05. *P*-values are based on Wilcoxon signed-rank tests and were not corrected for the multiplicity of testing.

4 NA = not applicable

***Phylum-level composition***. Vitamin B2 + C caused a significant decrease in *Proteobacteria* (*p* = .021) and displayed a trend toward increasing *Firmicutes* and decreasing *Bacteroidetes*, compared to the baseline (*p* = .052 and .064, respectively; Table 1). Moreover, with vitamin D, there was a trend for a decrease in *Bacteroidetes* when compared to baseline (*p* = .052) which was significant compared to the placebo (*p* = .038, Figure 2). Finally, vitamin D as well as vitamin A significantly increased *Actinobacteria* when compared to the placebo (*p*= .033 and *p* = .041, respectively; Figure 2).

***Genus-level composition.*** Vitamin supplementation caused significant changes in the relative abundance of *Alistipes, Bilophila, Clostridium, Collinsella*, an unidentified genus belonging to the family *Peptostreptococcaceae, Sutterella, Faecalibacterium, Coprococcus*, and *Odoribacter*, either when compared with the baseline within groups (Table 1) and/or when compared with the placebo group (Figure 2). Vitamin B2 + C induced a significant increase in *Coprococcus* (*p* = .027), and a significant decrease in *Sutterella* (*p* = .006) when compared with the baseline (Table 1); the latter effect was also evident when compared with the placebo group (*p* = .012; Figure 2). Moreover, vitamin B2 caused a significant increase in *Alistipes* and *Clostridium* when compared to the placebo (*p* = .015 and *p* = .004, respectively) while there was a decrease in *Faecalibacterium* abundance when compared with baseline (*p* = .012) (Table 1, Figure 2). Notably, all vitamins except vitamin B2 and E significantly increased *Collinsella* compared to the placebo (Figure 2). Vitamin treatments did not significantly affect the relative abundance of pathogens – including *Streptococcus, Enterobacter, Escherichia, Klebsiella, Providencia*, and *Shigella* – either within groups or between treatment and placebo groups **(Fig. S1)**.

***Species-level composition***. There were several changes with vitamin D including increases in *Bifidobacterium longum* and *Coprococcus comes* which were significant when compared to both the baseline and the placebo (*p* < .05, respectively, Table 1 and Figure 2). Moreover, when compared to the placebo, there was an increase in *Eubacterium hallii* (*p* = .038, Figure 2). In contrast, vitamin B2 decreased *E. hallii* and *Faecalibacterium prausnitzii* compared to the baseline (*p* = .042 and *p* = .012); however, this decrease was not significant when compared to the placebo (Table 1, Figure 2). All vitamins except B2 consistently increased *Collinsella aerofaciens* when compared to the placebo (Figure 2).

#### Colon-delivered vitamin C increases metabolic activity and reduces fecal pH and redox potential

**Figure 3.**
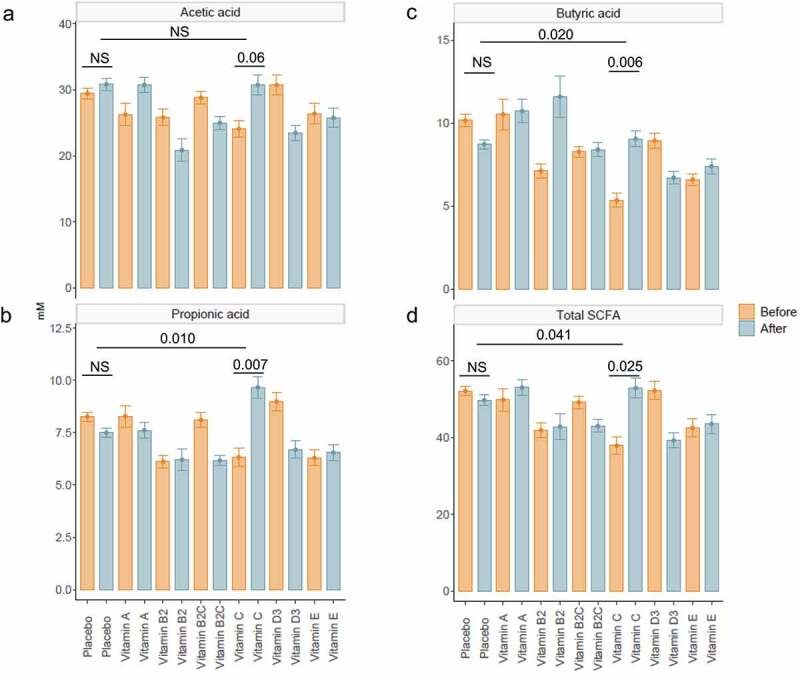
**Short-chain fatty acid concentrations before and after colon-delivered vitamin intervention**. Concentrations (mM) of acetate (a), propionate (b), butyrate (c) and total SCFA (d) before and after colon-delivered vitamin intervention were compared using the paired t-test when parametric assumptions were met, or a paired Wilcoxon test when parametric assumptions were not met. Absolute changes between the intervention group and the placebo were compared using the t-test when parametric assumption was met, or a Wilcoxon test when parametric assumptions were not met. Values are shown as mean ± SEM. NS, *p* > .05

Metabolic activity of the gut microbiome was assessed by measuring SCFA concentrations in the fecal content (Figure 3). We found that vitamin C significantly increased total SCFA (*p* = .025), as well as propionate (*p* = .007) and butyrate concentrations (*p* = .006) when compared with baseline. These effects were also evident when compared with that of the placebo (*p* = .041, *p* = .010 and *p* = .020, respectively).

Fecal pH was decreased more with vitamins C than with placebo, however, there was no significant difference between groups. Interestingly, fecal redox potential was decreased with vitamin C but increased with placebo; however, there was also no significant difference between groups **(Fig. S2)**.

#### Colon-delivered vitamin B2 increases fecal vitamin concentrations

To further confirm whether vitamins were delivered to the colon, we measured fecal and plasma concentrations of vitamin B2 before and after the intervention and compared with the placebo. Vitamin B2 in feces was increased when compared with baseline (*p* = .06) as well as with placebo (*p* = .05). However, there was no significant effect on plasma vitamin B2 concentrations **(Fig. S3)**.

#### Questionnaire data, safety and clinical parameters

There was no significant effect on the quality of life or gastrointestinal health scores **(Table S4)** and no adverse events, including hematology and biochemistry parameters were reported. Interestingly, there was a significant reduction in total fasting cholesterol with vitamin D when compared with baseline (*p* = .04) **(Table S1)**.

## In vitro study

### Effects of vitamins on gut microbial composition

**Figure 4.**
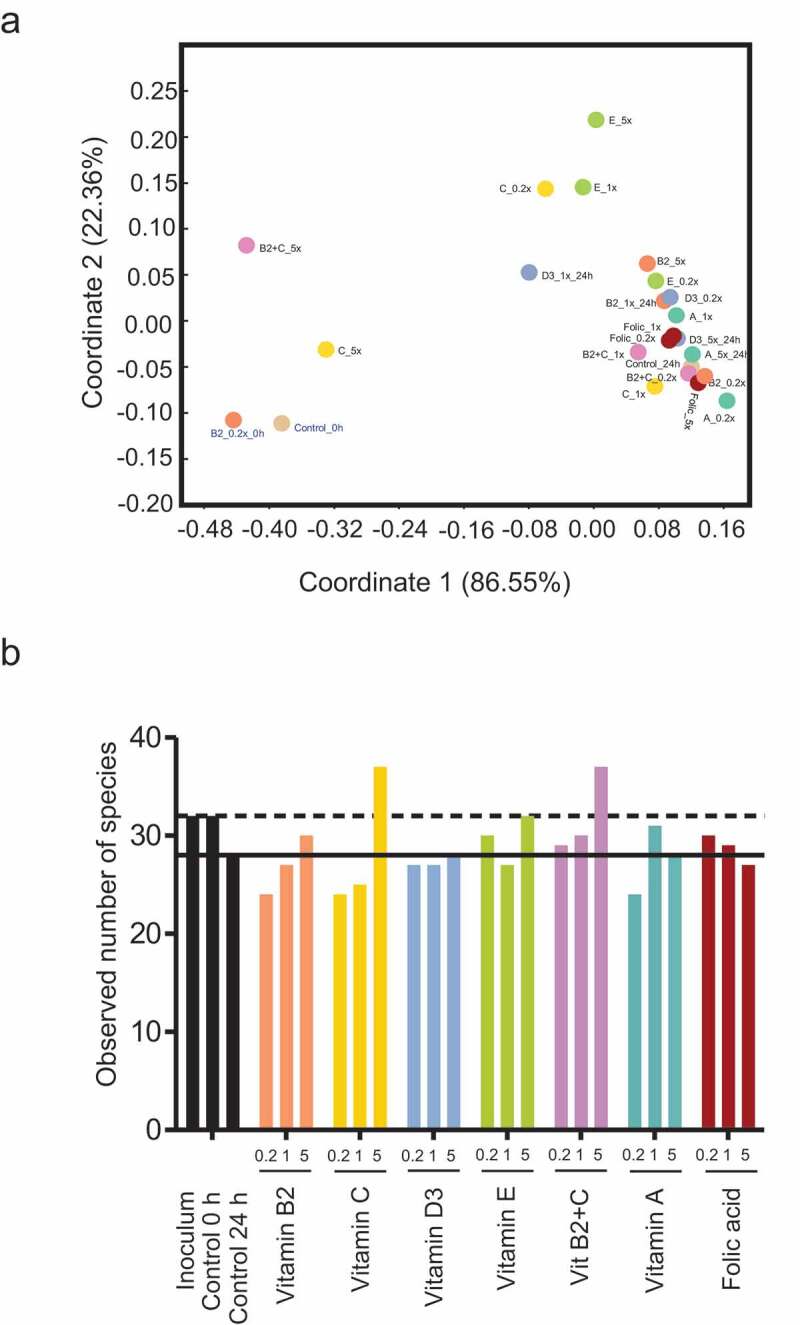
**Vitamin treatments induced changes in the composition of the gut microbiome in** **vitro.** (a) Non-metric multidimensional scaling (nMDS) analysis of microbiome profiles generated via fermentation supernatant samples. An additional sample was taken from vitamin B2 0.2x fermentation vessel at baseline to assess the consistency of microbiome profiling procedure. (b) The number of species in fermentation supernatant samplesEach vitamin was tested at 3 doses (0.2x, 1x, and 5x) **(Table S2) .**

Principle coordinate analysis (PCoA) on weighted UniFrac distances showed separation between before (0 h) and after (24 h) fermentation samples, indicating the effect of fermentation over time (Figure 4a). The majority of the microbiota treated with vitamins for 24 h clustered together with the 24 h control sample, indicating no change in beta diversity between vitamins and the control sample. However, 24 h samples treated with vitamin E at 1x and 5x, vitamin C at 0.2x and 5x, vitamin D at 1x, and vitamin B2 + C at 5x concentrations separated clearly from the 24 h control sample, suggesting that vitamin treatments had an effect on the composition of the microbiome during the fermentation process. Moreover, the addition of vitamin C, E, B2 + C, B2, A, and folic acid at various concentrations resulted in an increase in the observed number of species when compared with that of the control at 24 h, indicating an effect on alpha diversity (Figure 4b).

Vitamin treatments also induced several changes in the composition of the microbiota at all taxonomic levels. Changes that were most distinct at the phylum level were increases in *Actinobacteria, Firmicutes*, and *Verrucomicrobia* and decreases in Bacteroidetes particularly with vitamins E, B2, B2 + C and C **(Table S5)**. At the genus level, all vitamins except vitamin B2 induced a slight but consistent increase in *Roseburia* while vitamin C, B2, B2 + C, D and E, increased the relative abundance of *Akkermansia, Bifidobacterium* or Faecalibacterium **(Table S5)**.

We observed a number of consistent patterns when comparing *in vitro* data with the findings in humans. The increase in *Actinobacteria* and the decrease in *Bacteroidetes* were consistent with what was observed in humans vs. placebo (Figure 4). Similarly, the consistent effect on *Bifidobacterium* was largely in line with the effects in humans. Interestingly, the distinct effects of the majority of vitamins on *Coriobactericeae, Collinsella* and the species *Collinsella aerofaciens* in humans was evident in *in vitro* only with vitamin B2 + C.

### Effect of vitamins on metabolic activity of the gut microbiome

**Figure 5.**
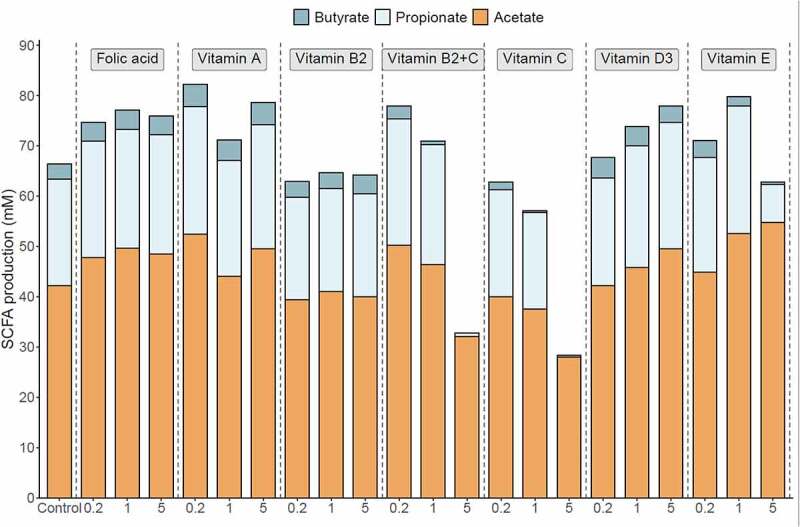
**Vitamin treatments induced changes in the metabolic activity of the gut microbiome in** **vitro.** SCFA production after 48 h fermentation upon/after addition of vitamins. Data are expressed as mM. Each vitamin was tested at 3 doses (0.2x, 1x, and 5x) **(Table S2) .**

The majority of vitamins induced a pronounced effect on SCFA production when compared with the control (Figure 5). Vitamin A treatment resulted in the highest total SCFA concentration (at 0.2x) while all concentrations of folic acid consistently increased total SCFAs.

Vitamin E treatment yielded the highest acetate concentration at the end of the 48 h fermentation process (at 5x) when compared with that of the control. Moreover, vitamin E (at 0.2x and 1x), vitamin A (at all concentrations), folic acid (at all concentrations), vitamin B2 + C (at 0.2x and 1x) and vitamin D (at 1x and 5x) led to higher acetate production, compared with that of the control.

The highest propionate concentrations were obtained with vitamin E (at 1x) and vitamin A (at 0.2x) when compared with that of the control treatment. Moreover, vitamin E (at 0.2x), vitamin D (at 1x and 5x), vitamin A (at 1x and 5x), vitamin B2 + C (at 0.2x and 1x) and folic acid (at all concentrations) increased propionate above that obtained from the control.

Vitamin A yielded the highest butyrate level (at all concentrations) compared to the control. Moreover, folic acid (at all concentrations), vitamin D (at all concentrations), vitamin B2 (at 5x), and vitamin E (at 0.2x) increased butyrate concentration when compared with the control sample.

Lactate concentration, gas production, and pH provide an additional measure of metabolic activity in *in vitro* fermentation systems **(Fig. S4)**. A marked decrease in pH, gas production, and lactate accumulation was observed following the addition of vitamin C at 1x and 5x, and vitamin E at 5x.

### Effects of vitamin-driven microbial metabolites on immunological biomarkers and gut barrier integrity

**Figure 6.**
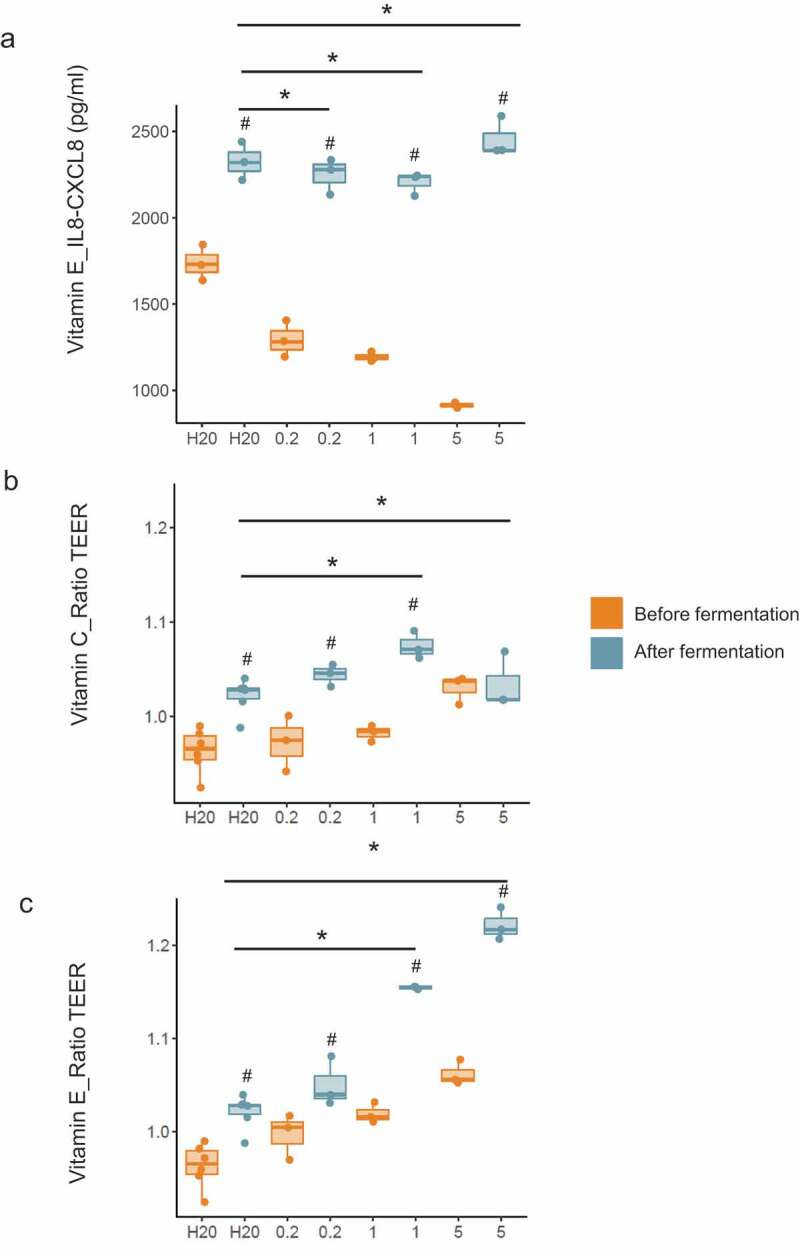
**Vitamin treatments improved immune function and barrier integrity *in vitro***. (a) Effect of vitamin E on IL-8-CXCL8 production by HT29 cells. Data are expressed as pg/mL. (b-c) Effects of vitamins C and E on gut barrier integrity using a cellular intestinal model. Data are expressed as the ratio between TEER at the end of the incubation period and initial TEER. TEER ratios and IL-8-CXCL8 concentrations of samples taken before and after fermentation were compared using unpaired t-tests and unpaired Wilcoxon tests. Absolute changes between vitamin treatment groups and the control (between reactors) were compared using a linear model. Values are shown as median and interquartile range. # = *p* < .05; before fermentation group; * = *p* < .05; control. Each vitamin was tested at 3 doses (0.2x, 1x, and 5x) **(Table S2)**

Various changes were observed in the secretion of GROa-CXCL1, IL8-CXCL8, and MIP3a-CCL20 when HT29 cells were incubated with fermentation samples of vitamin-treated microbiota (**Table S6**). A slight but consistent upregulation of IL8-CXCL8 (956.67 pg/mL, 1010.00 pg/mL, 1544.00 pg/mL) was evident at all concentrations of vitamin E when compared to the control (590.00 pg/mL) (*p* < .05) (Figure 6a). Moreover, all vitamin fermentation samples slightly reduced MIP3a-CCL20 levels when compared with the control (**Table S6**).

Regarding gut barrier integrity, we found that prior to fermentation, most vitamins increased TEER in a dose-dependent manner. Moreover, TEER was significantly increased in cells treated with control (water) supernatants following fermentation, and this effect was consistent throughout all experiments (Figure 6b-c for vitamin C and E; other vitamins not shown). An additional increases in TEER was observed for vitamin C at 1x and vitamin E at 1x and 5x and that was higher than that observed for the respective control samples (*p* < .05) (Figure 6b-c**)**.

## Discussion

The current study investigated the effects of colon-delivered vitamins on the human gut microbiota (HGM) in a clinical study and in short-term batch fermentation experiments which were combined with *in vitro* cell models to assess effects on the barrier and immune function. Our data demonstrate that particularly vitamins C, B2, and D modulate the HGM by altering its metabolic activity and/or bacterial composition. In humans, the effect was most distinct with vitamin C which significantly increased microbial alpha diversity and fecal SCFA when compared with placebo. Our *in vitro* data support these findings with several vitamins showing an effect on microbial diversity, composition, and/or metabolic activity, as well as trends for effects on host barrier and immune function. We used a colon-targeted delivery systems (CTDS) because under physiological conditions, vitamins do not reach the ileocolonic region but are efficiently absorbed in the proximal small intestine. Our data are in line with previous studies suggesting a direct effect of vitamins on the HGM either when overdosed or directly delivered to the large intestine.^27–30^

In humans, vitamin C increased bacterial evenness, fecal SCFAs including butyrate and propionate and the relative abundance of *Collinsella*. Our *in vitro* data are in line with this, showing an effect on bacterial alpha and beta diversity and increases in *Collinsella* at high dose vitamin C. Although *Collinsella*, the dominant taxon within the family *Coriobacteriaceae* has been linked in some studies to type 2 diabetes and increased levels of serum cholesterol,^31,32^ there is several other studies suggesting beneficial effects of this genus.^31,33^ For example, Delzenne and colleagues found that prebiotics such as inulin-type fructans increased *Collinsella* in obese women and that this correlated with higher urinary levels of Hippurate, a gut-derived metabolite commonly associated with a ‘healthy phenotype’ which is decreased in diabetes and obesity.^33^ In line with this, *Coriobacteriaceae* was recently found to be a potential contributor to the beneficial effects of Roux-en-Y gastric bypass on type 2 diabetes.^31^ Moreover, *Collinsella aerofaciens* has been associated with a low risk of colon cancer, and patients with IBD show lower gut levels of this genus than do control individuals.^34,35^ Interestingly, although *Collinsella spp* has been known traditionally to produce primarily formate and lactate, more recently, a novel butyrate-producing subspecies has been isolated from the human gut.^36,37^ This may explain part of the observed increases in fecal SCFA with vitamin C in humans.

*In vitro*, vitamin C also increased *Roseburia, Faecalibacterium, Akkermansia*, and *Bifidobacterium*, however, this was not evident in humans. Moreover, the low *in vitro* production of SCFAs with increasing concentrations of vitamin C contrasted our findings in humans. We believe that these differences are caused by limitations of the *in vitro* system primarily the lack of pH buffering capacity. In fact, there was a distinct pH drop with vitamin C and this was related to a decrease in overall metabolic activity reflected also by low gas production and accumulation of lactate. Contrarily, in both treatments with vitamin C (vitamin C and vitamin C + B2), we observed an increase in the observed number of species which seemed to contradict a decrease in overall metabolic activity. We assume that this observation is a result of the increased number of *Firmicutes* and *Actinobacteria* since gram positive bacteria have been shown to favor acidic pH conditions.^38,39^ On the other hand, the same mechanism may explain the observed decrease in *Bacteroidetes*, a gram negative bacteria.

Fecal pH and redox potential was reduced with vitamin C also in humans, and albeit not significant, we assume this may have contributed to the beneficial effects seen with vitamin C in the clinical study. In fact, the link between redox potential, oxidative stress and the HGM according to oxygen tolerance of each species and the abundance of antioxidants in the environment is meanwhile well established.^21,40–42^ For example, Million et al. presented data in humans linking the fecal redox potential to the ratio of aerotolerant versus strictly anaerobic species.^21,41^

B-vitamins, including riboflavin, may directly stabilize gut bacterial populations. In humanized gnotobiotic mice and *in vitro* anaerobic fecal cultures, the exchange and sharing of B-vitamins such as riboflavin contributed strongly to the maintenance of gut bacterial populations, with particular reference to auxotrophic species.^43^ It was hypothesized that at least some B-vitamin prototrophs must possess a general capacity to secrete B-vitamins into the extracellular milieu that are subsequently taken up by auxotrophic species, thus, ensuring their maintenance in the community. This hypothesis was substantiated by a recent study that systematically assessed the genomes of 256 common human gut bacteria for the presence of B-vitamin biosynthesis pathways.^44^ The authors reported an inverse pattern of vitamin syntheses, suggesting symbiotic relationships among gut microbiota organisms.^44^ Finally, a recent *in silico/in vitro* study elegantly demonstrated the dependency of the most abundant butyrate-producing *Firmicutes* species upon vitamins supplied from the diet or via cross-feeding.^45^ Interestingly, microbe-mediated B-vitamin production is reduced in type 2 diabetes, malnutrition and active CD patients;^46–48^ therefore, colonic supplementation may be a useful approach to counteract gut microbial dysbiosis.

In support of a modulatory effect of B-vitamins on the HGM, we found riboflavin to increase the observed number of species in both, the clinical study and *in vitro*. Given that low microbial diversity has been linked to antibiotic use and high-fat diets^49,50^ and several pathological conditions, including obesity and inflammatory bowel disease (IBD)^51,52^ colon-delivered riboflavin may, thus, be a useful approach to prevent and/or treat these conditions.

In contrast to earlier observations,^4,53–55^ we have not found riboflavin to increase *F. prausnitzii* which is in line with a more recent study in IBD patients supplemented with high doses (100 mg) of riboflavin over 3 weeks.^5^ However, riboflavin increased *Clostridium* and *Alistipes* which have been linked previously to host health.^56,57^

Lipid-soluble vitamins are not naturally produced by gut bacteria and their direct effects on the HGM employing CTDS or overdosing remain largely unexplored. In one study in healthy volunteers, high dose vitamin D reduced the relative abundance of *Gammaproteobacteria* and increased bacterial richness in the upper but not lower gastrointestinal tract.^58^ In addition, vitamin E dietary intake was found to correlate positively with Firmicutes and negatively with Bacteroidetes in free-living adults with cystic fibrosis.^30^ Our findings provide additional evidence for an effect of lipid-soluble vitamins particularly vitamin D and E on the HGM via direct colonic mechanisms. With vitamin D, we observed changes in several bacterial taxa including *Actinobacteria, Bifidobacterium* and *Bifidobacterium longum* both in humans and *in vitro*. Moreover, vitamin D led to an increase in *E. hallii*, a key species within the intestinal trophic chain with potential to impact metabolic balance, as well as gut microbiota/host homeostasis.^59^ Finally, vitamin D (as well as vitamins B2 + C) increased *Coprococcus*, a genus that was recently shown to be depleted in people with depression and associated with a higher quality of life.^60^ Furthermore, vitamin E increased the production of SCFAs as well as the relative abundance of *Akkermansia* and other beneficial microbes including *Lactobacillus, Bifidobacterium*, and *Faecalibacterium*. The effect on *Akkermansia* confirms recent observations in mice consuming high doses of vitamin E (0.18 mg/20 g of body weight per day) showing an increase in *Verrucomicrobia*, corresponding to those of *A. muciniphila* at the species level.^61^ Finally, vitamins E, D and A also increased *Coriobacteriaceae, Collinsella* and *Collinsella aerofaciens* as was observed for vitamin C suggesting a beneficial effect on host health. It is possible that some of the effects of vitamin D on the HGM are related to activation of the vitamin D receptor (VDR) given the VDR gene has been identified as a vital host factor that shapes the gut microbiome at the genetic level.^62^ Vitamin E, similar to vitamin C, may exert its effects by acting as an antioxidant to improve intestinal redox balance as mentioned earlier. We also found that vitamin C and E induced a dose-dependent increase in transepithelial electrical resistance (TEER), a well-known quantitative method to measure barrier integrity in cell culture models, where higher TEER indicates a tighter barrier. TEER increases were evident in samples before fermentation indicating that vitamins itself exerted a direct beneficial effect on barrier function which is in line with earlier studies.^63–65^ Moreover, vitamins C and E caused an additional increase in TEER over that observed in the respective control samples, indicating that vitamin fermentation supernatant a direct effect on gut barrier integrity. This result is intriguing given that SCFAs were reduced with increasing concentrations of vitamins C and E and suggests that other unmeasured metabolites produced during the fermentation process improved barrier functions independent of SCFAs. It also requires consideration that SCFA levels do not always correlate with TEER measurements^66,67^ which may explain the effects seen with folic acid that caused a consistent increase in SCFA but no barrier effects. Of note, intestinal barrier defects have been associated with a broad range of diseases, such as IBD, colon carcinoma, type 1 diabetes and obesity.^68^

Our results also showed a consistent upregulation of IL8-CXCL8 with vitamin E, suggesting that alpha tocopherol may exert an immunomodulatory effect. IL8 acts as a chemo-attractant of neutrophils, the recruitment of which constitutes an important early step in controlling tissue infections or injury.^69,70^ It may be useful to investigate whether this effect continues under challenging conditions, such as co-treatment with TNF-α.

The current study did not observe any adverse events; moreover, there was no significant effect on either the quality of life or gastrointestinal health scores, although some trends, such as an increase in emotional well-being with vitamin A and E were observed. Interestingly, we also found that colon-delivered vitamin D significantly reduced total fasting cholesterol when compared to baseline; however, these findings warrant a more comprehensive investigation to draw meaningful conclusions. Importantly, colon-delivered vitamins did not enhance the growth of pathogens, which is substantiated by numerous *in vitro* and animal studies suggesting enhanced pathogen clearance.^58,71–73^

Our study was restricted by several limitations that require consideration. 1) A small sample size allowed only a limited interpretation of results due to a lack of statistical power. 2) Diet is a key modulator of the gut microbiota. Although there was no difference in participants’ dietary intake at baseline **(Table S7)**, there was no dietary monitoring during the intervention and hence changes in habitual diet may have influenced the outcome. 3) Some studies have contended that the reliability of the delivery system used in the current study is limited in nature.^74^ Thus, the use of advanced technologies to ensure colon delivery may produce more pronounced effects. In order to confirm colonic delivery, we measured fecal and plasma concentrations of vitamin B2, and observed an increased fecal concentration of riboflavin, while plasma levels remained unchanged. This suggested that vitamin B2 was delivered to the colon, but not readily absorbed from the colon. However, our study was not designed to investigate pharmacokinetics of the delivery system. 4) The *in vitro* gut cellular models lacked biological replication, although this was partially compensated by using three different doses it only allows limited interpretation.

In conclusion, the current study presents pilot data indicating that colon-delivered vitamins exert a modulatory effect on the human gut microbiome and related metabolic activity including the production of SCFA. Based on effects seen in both humans and *in vitro*, vitamins C, B2, and D appear to be the most promising among the vitamins tested. However, other vitamins such as vitamin E warrant further investigation as well as HGM related effects on host immune and barrier functions. In addition, further research is required to explore the significance of adopting this novel concept for clinical application. This would include the treatment and prevention of human diseases – such as type 2 diabetes, cardiovascular disease, cancer, depression, and Parkinson’s disease – that have been linked to microbiota dysbiosis, which is largely attributed to a modern lifestyle.

## Supplementary Material

Supplemental Material
