# Supplementary material for: Effects of colon-targeted vitamins on the composition and metabolic activity of the human gut microbiome– a pilot study
Source: Gut Microbes. 2021 Feb 21;13(1):1875774. doi: 10.1080/19490976.2021.1875774 (PMC7899684; doi:10.1080/19490976.2021.1875774)
Supplement: Supplemental Material [file KGMI_A_1875774_SM7459.zip › Supplementary information/Additional file 1_revised.docx]

**Table S1. Subject characteristics and clinical parameters**

|  | **Vitamin A**  **(n=12)** | **Vitamin B2**  **(n=12)** | **Vitamin C**  **(n=12)** | **Vitamin B2 +C**  **(n=12)** | **Vitamin D**  **(n=12)** | **Vitamin E**  **(n=12)** | **Placebo**  **(n=24)** |
| --- | --- | --- | --- | --- | --- | --- | --- |
| **Age, years**^1,2^ | 37.5 (2.6) | 39.33 (2.11) | 34.42 (2.11) | 41.50 (2.22) | 39.50 (2.45) | 33.83 (2.18) | 36.50 (1.62) |
| **Male/Female (n)** | 7/5 | 4/8 | 7/5 | 5/7 | 6/6 | 6/6 | 13/11 |
| **Fiber consumption, g/day**^1,2^ | 22.8 (1.9) | 22.5 (1.7) | 22.7 (1.7) | 21.5 (2.1) | 22.1 (1.9) | 21.7 (1.3) | 21.8 (1.4) |
| **BMI, kg/m^2^** ^1,2^ |  |  |  |  |  |  |  |
| Before | 25.68 (0.87) | 25.27 (0.68) | 24.75 (0.74) | 23.89 (0.86) | 24.40 (0.97) | 25.29 (0.62) | 25.23 (0.53) |
| After | 25.29 (0.84) | 25.06 (0.70) | 24.65 (0.71) | 23.87 (0.87) | 24.54 (0.98) | 25.14 (0.60) | 25.27 (0.55) |
| P values^3^ | 0.11 | 0.06 | 0.36 | 0.85 | 0.23 | 0.52 | 0.55 |
| **Diastolic Blood Pressure (mm Hg)** ^1,2^ |  |  |  |  |  |  |  |
| Before | 72.83 (3.55) | 76.500 (3.31) | 77.167 (3.90) | 71.250 (2.31) | 78.083 (2.12) | 74.667 (3.48) | 78.125 (2.28) |
| After | 73.92 (3.67) | 76.08 (3.05) | 75.08 (3.29) | 68.33 (3.16) | 75.42 (2.64) | 76.33 (3.46) | 76.79 (2.06) |
| P values | 0.67 | 0.86 | 0.51 | 0.33 | 0.24 | 0.51 | 0.36 |
| **Systolic Blood Pressure (mm Hg)** ^1,2^ |  |  |  |  |  |  |  |
| Before | 114.08 (4.22) | 115.750 (4.91) | 120.250 (4.44) | 109.583 (3.99) | 114.583 (3.71) | 118.500 (4.03) | 118.083 (3.16) |
| After | 121.75 (5.10) | 115.08 (3.87) | 117.25 (4.06) | 108.83 (3.97) | 115.42 (3.14) | 118.08 (3.75) | 117.71 (3.33) |
| P values | 0.06 | 0.82 | 0.28 | 0.75 | 0.70 | 0.89 | 0.86 |
| **Cholesterol,Fasting (mmol/L)** ^1,2^ |  |  |  |  |  |  |  |
| Before | 4.617 (0.33) | 4.892 (0.27) | 4.675 (0.22) | 4.725 (0.19) | 4.667 (0.25) | 4.450 (0.15) | 4.821 (0.18) |
| After | 4.80 (0.32) | 5.02 (0.29) | 4.53 (0.21) | 4.55 (0.21) | 4.50 (0.28) | 4.62 (0.14) | 4.85 (0.19) |
| P values | 0.29 | 0.29 | 0.20 | 0.28 | 0.04 | 0.06 | 0.29 |
| **Cholesterol,HDL (mmol/L)** ^1,2^ |  |  |  |  |  |  |  |
| Before | 1.29 (0.09) | 1.58 (0.13) | 1.44 (0.09) | 1.68 (0.12) | 1.61 (0.12) | 1.58 (0.10) | 1.52 (0.07) |
| After | 1.26 (0.08) | 1.56 (0.10) | 1.36 (0.09) | 1.59 (0.14) | 1.50 (0.10) | 1.55 (0.11) | 1.46 (0.06) |
| P values | 0.42 | 0.72 | 0.17 | 0.17 | 0.07 | 0.39 | 0.16 |
| **Cholesterol,LDL (mmol/L)** ^1,2^ |  |  |  |  |  |  |  |
| Before | 2.85 (0.30) | 2.84 (0.27) | 2.75 (0.19) | 2.62 (0.15) | 2.59 (0.22) | 2.46 (0.11) | 2.81 (0.17) |
| After | 3.02 (0.29) | 2.99 (0.29) | 2.65 (0.18) | 2.59 (0.16) | 2.52 (0.22) | 2.65 (0.12) | 2.91 (0.17) |
| P values | 0.28 | 0.15 | 0.27 | 0.68 | 0.18 | 0.07 | 0.37 |
| **CRP hs (mg/L)** ^1,2^ |  |  |  |  |  |  |  |
| Before | 1.78 (0.57) | 1.09 (0.17) | 1.38 (0.41) | 1.14 (0.26) | 1.48 (0.51) | 1.61 (0.57) | 1.892 (0.57) |
| After | 2.71 (1.29) | 1.12 (0.27) | 1.30 (0.38) | 1.88 (0.43) | 1.23 (0.28) | 2.17 (0.69) | 1.58 (0.36) |
| P values | 0.38 | 0.90 | 0.58 | 0.138 | 0.438 | 0.437 | 0.63 |
| **Glucose,Fasting (mmol/L)** ^1,2^ |  |  |  |  |  |  |  |
| Before | 4.792 (0.29) | 4.675 (0.10) | 4.783 (0.12) | 4.700 (0.14) | 4.736 (0.07) | 4.418 (0.08) | 4.779 (0.09) |
| After | 4.80 (0.28) | 4.65 (0.15) | 4.67 (0.09) | 4.61 (0.11) | 4.65 (0.08) | 4.41 (0.10) | 4.72 (0.09) |
| P values | 0.69 | 0.85 | 0.22 | 0.67 | 0.34 | 0.84 | 0.41 |
| **Triglyceride,Fasting (mmol/L)**^1,2^ |  |  |  |  |  |  |  |
| Before | 1.042 (0.16) | 1.058 (0.11) | 1.042 (0.22) | 0.908 (0.07) | 1.000 (0.11) | 0.867 (0.14) | 1.054 (0.08) |
| After | 1.10 (0.15) | 1.04 (0.08) | 1.17 (0.28) | 0.98 (0.06) | 1.09 (0.12) | 0.95 (0.09) | 1.06 (0.08) |
| P values | 0.57 | 0.86 | 0.30 | 0.46 | 0.26 | 0.24 | 0.90 |
| **Urea (mmol/L)**^1,2^ |  |  |  |  |  |  |  |
| Before | 4.43 (0.27) | 4.38 (0.25) | 5.15 (0.29) | 4.91 (0.34) | 4.82 (0.35) | 4.97 (0.35) | 4.59 (0.22) |
| After | 4.59 (0.31) | 5.02 (0.39) | 5.09 (0.42) | 5.47 (0.35) | 4.93 (0.39) | 4.97 (0.26) | 4.77 (0.20) |
| P values | 0.57 | 0.05 | 0.86 | 0.14 | 0.67 | 0.97 | 0.44 |
| **Uric Acid (µmol/L)**^1,2^ |  |  |  |  |  |  |  |
| Before | 298.67 (24.13) | 273.67 (22.92) | 308.58 (21.12) | 286.58 (14.48) | 269.00 (18.02) | 301.00 (24.69) | 307.00 (14.63) |
| After | 290.33 (22.41) | 266.36 (22.06) | 298.17 (18.49) | 283.08 (14.74) | 269.75 (18.88) | 293.00 (29.20) | 304.04 (12.79) |
| P values | 0.39 | 0.46 | 0.28 | 0.66 | 0.92 | 0.57 | 0.74 |

1. Data are shown as mean ± SEM
2. There was no statistically significant difference between groups using ANOVA
3. Within group vs. baseline using paired t-test
