# Supplementary material for: Effects of colon-targeted vitamins on the composition and metabolic activity of the human gut microbiome– a pilot study
Source: Gut Microbes. 2021 Feb 21;13(1):1875774. doi: 10.1080/19490976.2021.1875774 (PMC7899684; doi:10.1080/19490976.2021.1875774)
Supplement: Supplemental Material [file KGMI_A_1875774_SM7459.zip › Supplementary information/Additional file 2.docx]

|  | Dose | Final concentration |
| --- | --- | --- |
| Vitamin B2 (riboflavin) | 0.2x | 0.06 mg/ml |
|  | 1x^1^ | 0.29 mg/ml |
|  | 5x | 1.43 mg/ml |
| Vitamin C (ascorbic acid) | 0.2x | 0.43 mg/ml |
|  | 1x | 2.14 mg/ml |
|  | 5x | 10.71 mg/ml |
| Vitamin E (alpha-Tocopherol) | 0.2x | 0.07 mg/ml |
|  | 1x | 0.36 mg/ml |
|  | 5x | 1.79 mg/ml |
| Vitamin D3 (cholecalciferol) | 0.2x | 0.04 µg/ml |
|  | 1x | 0.21 µg/ml |
|  | 5x | 1.07 µg/ml |
| Vitamin A (retinol) | 0.2x | 0.18 µg/ml |
|  | 1x | 0.90 µg/ml |
|  | 5x | 4.52 µg/ml |
| Vitamin B9 (folic acid) | 0.2x | 0.29 µg/ml |
|  | 1x | 1.43 µg/ml |
|  | 5x | 7.14 µg/ml |
| Vitamin B2 + Vitamin C | 0.2x | 0.06 mg/ml riboflavin and 0.43 mg/ml ascorbic acid |
|  | 1x | 0.29 mg/ml riboflavin and 2,14 mg/ml ascorbic acid |
|  | 5x | 1,43 mg/ml riboflavin and 10,71 mg/ml ascorbic acid |

**Table S2. Dose designation and final concentration of micronutrients in *in vitro***

**fermentation experiments**

1 1x dose was determined by subtracting high dose oral delivery of vitamins in previous studies [1–5] by the estimated intestinal absorption level for each vitamin [6–9].

1. Steinert RE, Sadaghian SM, Harmsen HJM, Weber P. The prebiotic concept and human health: a changing landscape with riboflavin as a novel prebiotic candidate? Eur J Clin Nutr**.** 2016;70:1461. doi:10.1038/ejcn.2016.141.

2. Tang M, Frank DN, Sherlock L, Ir D, Robertson CE, Krebs NF. Effect of vitamin E with therapeutic iron supplementation on iron repletion and gut microbiome in U.S. iron deficient infants and toddlers: a randomized control trial. J Pediatr Gastroenterol Nutr**.** 2016;63:379–85. doi:10.1097/MPG.0000000000001154.

3. de Vries APJ, Oterdoom LH, Gans ROB, Bakker SJL. Supplementation with anti-oxidants Vitamin C and E decreases cyclosporine A trough-levels in renal transplant recipients. Nephrol Dial Transplant. 2006;21:231–2. doi:10.1093/ndt/gfi112.

4. Cantarel BL, Waubant E, Chehoud C. Kuczynski J; DeSantis TZ; Warrington J; et al. Gut microbiota in MS: possible influence of immunomodulators. J Investig Med**.** 2015;63,729–34. doi:10.1097/JIM.0000000000000192.

5. Lakoff A, Fazili Z, Aufreiter S, Pfeiffer CM, Connolly B, Gregory JF, et al. Folate is absorbed across the human colon: evidence by using enteric-coated caplets containing 13C-labeled [6S]-5-formyltetrahydrofolate. Am J Clin Nutr. 2014*;*100:1278–86. doi:10.3945/ajcn.114.091785.

6. Basu TK, Donaldson D. Intestinal absorption in health and disease: micronutrients. Best Pract Res Clin Gastroenterol**.** 2003;17:957–79. doi:10.1016/s1521-6918(03)00084-2.

7. Reboul E. Absorption of Vitamin A and Carotenoids by the Enterocyte: Focus on Transport Proteins. Nutrients**.** 2013;5:3563–81. doi:10.3390/nu5093563.

8. Gropper S, Smith J, Groff JL. *Advanced Nutrition and Human Metabolism*. Cengage Learning, 2004; ISBN 978-0-534-55986-1.

9. Graf E. Vitamin E: A Comprehensive Treatise (Basic and Clinical Nutrition, Volume 1). In: Machlin LJ, editor. 660 S geb., illustriert, Sfr. 168, *Pharmazie in unserer Zeit.* New York: Marcel Dekker, Inc; 1980; p. 158.
