# Supplementary material for: Effects of colon-targeted vitamins on the composition and metabolic activity of the human gut microbiome– a pilot study
Source: Gut Microbes. 2021 Feb 21;13(1):1875774. doi: 10.1080/19490976.2021.1875774 (PMC7899684; doi:10.1080/19490976.2021.1875774)
Supplement: Supplemental Material [file KGMI_A_1875774_SM7459.zip › Supplementary information/Additional file 3.docx]

**Table S3. Permutational multivariate analysis of variance on Bray-Curtis distances in response to colon-delivered vitamins**

|  | **Before_Placebo** | **After_Placebo** | **Before_Vitamin A** | **After_Vitamin A** | **Before_Vitamin B2** | **After_Vitamin B2** | **Before_Vitamin B2+C** | **After_Vitamin B2+C** | **Before_Vitamin C** | **After_Vitamin C** | **Before_Vitamin D3** | **After_Vitamin D3** | **Before_Vitamin E** | **After_Vitamin E** |
| --- | --- | --- | --- | --- | --- | --- | --- | --- | --- | --- | --- | --- | --- | --- |
| **Before_Placebo** | 0 | 0.9981 | 0.1377 | 0.3915 | 0.2679 | 0.0046 | 0.0676 | 0.3207 | 0.6629 | 0.3858 | 0.3684 | 0.5335 | 0.0279 | 0.1583 |
| **After_Placebo** |  | 0 | 0.3411 | 0.8797 | 0.6201 | 0.0917 | 0.2441 | 0.6891 | 0.7873 | 0.8222 | 0.6126 | 0.7264 | 0.3501 | 0.7407 |
| **Before_Vitamin A** |  |  | 0 | 0.9946 | 0.4962 | 0.2415 | 0.8817 | 0.7472 | 0.4321 | 0.4283 | 0.4478 | 0.0787 | 0.0924 | 0.5495 |
| **After_Vitamin A** |  |  |  | 0 | 0.6214 | 0.3178 | 0.4059 | 0.8632 | 0.3016 | 0.4007 | 0.1443 | 0.2307 | 0.4525 | 0.7787 |
| **Before_Vitamin B2** |  |  |  |  | 0 | 0.8119 | 0.2456 | 0.3564 | 0.2657 | 0.2628 | 0.3063 | 0.1272 | 0.3615 | 0.7378 |
| **After_Vitamin B2** |  |  |  |  |  | 0 | 0.1898 | 0.1571 | 0.0617 | 0.0819 | 0.0719 | 0.0127 | 0.416 | 0.5096 |
| **Before_Vitamin B2+C** |  |  |  |  |  |  | 0 | 0.9878 | 0.4865 | 0.4748 | 0.4986 | 0.1774 | 0.0925 | 0.3838 |
| **After_Vitamin B2+C** |  |  |  |  |  |  |  | 0 | 0.5994 | 0.5358 | 0.4457 | 0.6217 | 0.2435 | 0.6724 |
| **Before_Vitamin C** |  |  |  |  |  |  |  |  | 0 | 0.992 | 0.9556 | 0.3393 | 0.0855 | 0.4535 |
| **After_Vitamin C** |  |  |  |  |  |  |  |  |  | 0 | 0.8765 | 0.2312 | 0.2044 | 0.6734 |
| **Before_Vitamin D3** |  |  |  |  |  |  |  |  |  |  | 0 | 0.7374 | 0.0455 | 0.3919 |
| **After_Vitamin D3** |  |  |  |  |  |  |  |  |  |  |  | 0 | 0.0186 | 0.079 |
| **Before_Vitamin E** |  |  |  |  |  |  |  |  |  |  |  |  | 0 | 0.9958 |
| **After_Vitamin E** |  |  |  |  |  |  |  |  |  |  |  |  |  | 0 |
