# Supplementary material for: Effects of colon-targeted vitamins on the composition and metabolic activity of the human gut microbiome– a pilot study
Source: Gut Microbes. 2021 Feb 21;13(1):1875774. doi: 10.1080/19490976.2021.1875774 (PMC7899684; doi:10.1080/19490976.2021.1875774)
Supplement: Supplemental Material [file KGMI_A_1875774_SM7459.zip › Supplementary information/Additional file 4.docx]

**Table S4. Changes in the quality of life and gastrointestinal health scores in response to colon-delivered vitamins**

|  | **SF-36^1^ Energy/Fatigue** | **SF-36 Emotional Well-Being** | **SF-36 General Health** | **GSRS^2^** |
| --- | --- | --- | --- | --- |
| **Placebo** |  |  |  |  |
| **Before** | 74.77 ± 3.11^3^ | 84.91 ± 2.43 | 83.41 ± 2.19 | 22.09 ± 0.97 |
| **After** | 74.09 ± 3.28 | 86.00 ± 2.24 | 85.00 ± 2.57 | 21.64 ± 0.94 |
| ***P* value**^4^ | 0.81/NA | 0.63/NA | 0.54/NA | 0.54/NA |
| **Vitamin A** |  |  |  |  |
| **Before** | 79.55 ± 4.69 | 85.09 ± 4.18 | 91.36 ± 2.79 | 20.09 ± 1.10 |
| **After** | 76.82 ± 2.96 | 91.27 ± 1.41 | 89.09 ± 3.00 | 20.73 ± 1.17 |
| ***P* value** | 0.61/0.99 | 0.09/0.89 | 0.42/0.94 | 0.43/0.99 |
| **Vitamin B2** |  |  |  |  |
| **Before** | 78.33 ± 3.28 | 87.67 ± 1.87 | 88.33 ± 2.78 | 21.33 ± 1.18 |
| **After** | 76.67 ± 4.19 | 86.00 ± 3.13 | 83.75 ± 3.70 | 21.50 ± 1.37 |
| ***P* value** | 0.58/0.99 | 0.61/0.99 | 0.06/0.61 | 0.96/0.99 |
| **Vitamin C** |  |  |  |  |
| **Before** | 65.83 ± 4.56 | 83.00 ± 4.41 | 82.08 ± 4.62 | 26.08 ± 3.11 |
| **After** | 61.67 ± 6.16 | 80.67 ± 4.79 | 84.58 ± 4.46 | 21.50 ± 1.37 |
| ***P* value** | 0.48/0.99 | 0.63/0.98 | 0.31/0.99 | 0.72/0.99 |
| **Vitamin B2+C** |  |  |  |  |
| **Before** | 71.50 ± 4.89 | 80.40 ± 5.58 | 87.50 ± 3.96 | 22.60 ± 1.59 |
| **After** | 71.50 ± 4.66 | 77.20 ± 5.60 | 90.50 ± 2.83 | 23.40 ± 2.00 |
| ***P* value** | 0.99/0.99 | 0.27/0.96 | 0.14/0.99 | 0.72/0.99 |
| **Vitamin D3** |  |  |  |  |
| **Before** | 73.18 ± 3.65 | 85.82 ± 2.77 | 84.55 ± 4.64 | 23.36 ± 2.31 |
| **After** | 66.82 ± 5.36 | 79.64 ± 4.62 | 82.27 ± 4.69 | 22.73 ± 2.36 |
| ***P* value** | 0.16/0.93 | 0.14/0.61 | 0.57/0.94 | 0.99/0.99 |
| **Vitamin E** |  |  |  |  |
| **Before** | 69.50 ± 3.98 | 86.00 ± 2.94 | 80.00 ± 4.83 | 23.00 ± 1.88 |
| **After** | 76.50 ± 2.48 | 85.60 ± 1.90 | 85.50 ± 3.53 | 22.10 ± 1.27 |
| ***P* value** | 0.08/0.78 | 0.88/0.99 | 0.15/0.97 | 0.72/0.99 |

1 The 36-Item Short Form Health survey questionnaire

2 Gastrointestinal Symptom Rating Scale

3 Data are shown as mean ± SEM

4 Within group vs. baseline using paired t-test or paired Wilcoxon test/between groups vs. placebo using t-test or Wilcoxon test
