# Supplementary material for: Effects of colon-targeted vitamins on the composition and metabolic activity of the human gut microbiome– a pilot study
Source: Gut Microbes. 2021 Feb 21;13(1):1875774. doi: 10.1080/19490976.2021.1875774 (PMC7899684; doi:10.1080/19490976.2021.1875774)
Supplement: Supplemental Material [file KGMI_A_1875774_SM7459.zip › Supplementary information/Additional file 5_revised.docx]

**Table S5. Changes in the composition of the gut microbiota at phylum and genus levels in response to vitamins *in vitro*.**

|  | **Control** | **Vitamin B2** | | | **Vitamin C** | | | **Vitamin E** | | | **Vitamin D3** | | | **Vitamin A** | | | **Vitamin B2+C** | | | **Folic acid** | | |
| --- | --- | --- | --- | --- | --- | --- | --- | --- | --- | --- | --- | --- | --- | --- | --- | --- | --- | --- | --- | --- | --- | --- |
| **Dose** | **-** | **0.2x^1^** | **1x** | **5x** | **0.2x** | **1x** | **5x** | **0.2x** | **1x** | **5x** | **0.2x** | **1x** | **5x** | **0.2x** | **1x** | **5x** | **0.2x** | **1x** | **5x** | **0.2** | **1** | **5** |
| ***Phylum*** |  |  |  |  |  |  |  |  |  |  |  |  |  |  |  |  |  |  |  |  |  |  |
| Actinobacteria | 10.9370 | 10.1642 | 16.5053 | 17.6021 | 21.6646 | 14.4735 | 13.2397 | 16.7731 | 23.8350 | 29.6327 | 15.3322 | 13.0836 | 12.8707 | 6.1253 | 13.4418 | 10.1185 | 10.6519 | 16.9352 | 15.0964 | 13.0355 | 13.0606 | 9.5722 |
| Bacteroidetes | 78.0894 | 79.6553 | 71.0609 | 67.7533 | 60.2904 | 79.0153 | 66.0934 | 69.2094 | 61.3700 | 61.1815 | 70.6452 | 66.7883 | 75.3024 | 82.7553 | 72.7064 | 77.4122 | 78.8353 | 75.4213 | 61.9944 | 74.7867 | 74.9140 | 79.7120 |
| Firmicutes | 7.8592 | 6.8389 | 9.2184 | 10.6483 | 12.7851 | 2.8293 | 16.5780 | 10.4155 | 9.5127 | 3.2695 | 10.8752 | 13.4395 | 8.3003 | 7.3336 | 10.5972 | 9.0484 | 7.2301 | 3.9564 | 15.1934 | 8.9520 | 8.7433 | 7.4801 |
| Proteobacteria | 3.0108 | 3.2651 | 3.0444 | 3.7289 | 5.0022 | 3.4830 | 2.3887 | 3.4608 | 5.0731 | 5.7549 | 3.0034 | 6.5354 | 3.4042 | 3.7031 | 3.1231 | 3.3205 | 3.1618 | 3.4769 | 6.3232 | 3.1204 | 3.1567 | 3.1229 |
| Verrucomicrobia | 0.1036 | 0.0765 | 0.1710 | 0.2674 | 0.2577 | 0.1989 | 1.7002 | 0.1412 | 0.2092 | 0.1614 | 0.1440 | 0.1532 | 0.1223 | 0.0828 | 0.1315 | 0.1004 | 0.1208 | 0.2102 | 1.3925 | 0.1054 | 0.1255 | 0.1127 |
| ***Genus*** |  |  |  |  |  |  |  |  |  |  |  |  |  |  |  |  |  |  |  |  |  |  |
| *Anaerostipes* | 0.1580 | 0.1675 | 0.2781 | 0.3785 | 0.2564 | 0.0038 | 0.0917 | 0.3304 | 0.2288 | 0.0213 | 0.4108 | 0.2755 | 0.2369 | 0.1421 | 0.3516 | 0.1920 | 0.0943 | 0.0175 | 0.0992 | 0.1581 | 0.1457 | 0.1365 |
| *Blautia* | 0.0768 | 0.0751 | 0.0535 | 0.0374 | 0.1420 | 0.0133 | 0.2022 | 0.0976 | 0.0930 | 0.1017 | 0.1174 | 0.1403 | 0.0625 | 0.0376 | 0.0906 | 0.0662 | 0.0665 | 0.0343 | 0.2060 | 0.1078 | 0.0563 | 0.0394 |
| *Clostridium* | 2.6854 | 2.6990 | 3.2781 | 3.4592 | 4.8086 | 0.6073 | 0.2073 | 3.4526 | 3.2525 | 0.8745 | 3.4050 | 4.3720 | 2.9250 | 2.2127 | 3.4068 | 3.1125 | 2.6250 | 0.8883 | 0.1687 | 2.9570 | 2.7030 | 2.3900 |
| *Collinsella* | 0.0002 | 0.0032 | 0.0000 | 0.0028 | 0.0000 | 0.0000 | 0.1398 | 0.0052 | 0.0000 | 0.0043 | 0.0000 | 0.0000 | 0.0000 | 0.0029 | 0.0072 | 0.0033 | 0.0012 | 0.0049 | 0.1434 | 0.0066 | 0.0027 | 0.0011 |
| *Coprobacillus* | 0.0093 | 0.0000 | 0.0064 | 0.0032 | 0.0093 | 0.0000 | 0.0000 | 0.0152 | 0.0058 | 0.0008 | 0.0026 | 0.0067 | 0.0036 | 0.0000 | 0.0137 | 0.0034 | 0.0000 | 0.0000 | 0.0000 | 0.0008 | 0.0000 | 0.0000 |
| *Coprobacter* | 0.1743 | 0.1514 | 0.1368 | 0.0830 | 0.3072 | 0.2446 | 0.7066 | 0.1423 | 0.2514 | 0.2396 | 0.1368 | 0.2919 | 0.1338 | 0.1894 | 0.1473 | 0.1618 | 0.1481 | 0.2298 | 0.6387 | 0.1661 | 0.1738 | 0.1842 |
| *Dialister* | 1.0401 | 1.1188 | 1.5223 | 1.9590 | 1.8956 | 0.4611 | 0.1884 | 1.4872 | 1.2885 | 0.1403 | 1.4434 | 1.7421 | 1.1552 | 0.9122 | 1.4338 | 1.2027 | 0.9889 | 0.6319 | 0.1752 | 1.2241 | 1.2255 | 1.0603 |
| *Dorea* | 0.2392 | 0.2012 | 0.1979 | 0.1245 | 0.3836 | 0.2223 | 0.3210 | 0.4264 | 0.9265 | 0.6704 | 0.4013 | 0.7479 | 0.4196 | 0.4662 | 0.3989 | 0.3746 | 0.2483 | 0.1842 | 0.2643 | 0.1604 | 0.2155 | 0.2449 |
| *Eggerthella* | 0.0582 | 0.0598 | 0.1180 | 0.2410 | 0.1300 | 0.1556 | 0.0774 | 0.0780 | 0.1026 | 0.1145 | 0.0774 | 0.0556 | 0.0931 | 0.0311 | 0.0641 | 0.0421 | 0.0718 | 0.1606 | 0.1507 | 0.0620 | 0.0741 | 0.0563 |
| *Eubacterium* | 0.0298 | 0.0129 | 0.0201 | 0.0201 | 0.0266 | 0.0148 | 0.5901 | 0.0194 | 0.0104 | 0.0358 | 0.0141 | 0.0254 | 0.0131 | 0.0134 | 0.0208 | 0.0044 | 0.0117 | 0.0384 | 0.4257 | 0.0152 | 0.0216 | 0.0188 |
| *Flavonifractor* | 0.0006 | 0.0000 | 0.0050 | 0.0098 | 0.0000 | 0.0000 | 0.0303 | 0.0012 | 0.0000 | 0.0073 | 0.0000 | 0.0000 | 0.0000 | 0.0000 | 0.0058 | 0.0000 | 0.0041 | 0.0036 | 0.0163 | 0.0051 | 0.0018 | 0.0106 |
| *Odoribacter* | 0.1228 | 0.0394 | 0.1815 | 0.0337 | 0.0186 | 0.3206 | 0.8383 | 0.2332 | 0.3985 | 0.3246 | 0.0111 | 0.4208 | 0.2420 | 0.0373 | 0.0475 | 0.2529 | 0.0378 | 0.3104 | 0.4286 | 0.0091 | 0.2266 | 0.2500 |
| *Roseburia* | 0.0025 | 0.0014 | 0.0027 | 0.0000 | 0.0056 | 0.0100 | 0.0419 | 0.0138 | 0.0331 | 0.0436 | 0.0090 | 0.0183 | 0.0078 | 0.0098 | 0.0086 | 0.0131 | 0.0050 | 0.0138 | 0.0375 | 0.0073 | 0.0071 | 0.0144 |
| *Streptococcus* | 0.0008 | 0.0000 | 0.0000 | 0.0000 | 0.0015 | 0.0000 | 0.2106 | 0.0000 | 0.0000 | 0.0004 | 0.0000 | 0.0000 | 0.0000 | 0.0000 | 0.0012 | 0.0000 | 0.0008 | 0.0048 | 0.1298 | 0.0000 | 0.0000 | 0.0000 |
| *Veillonella* | 2.1886 | 1.2776 | 2.3511 | 3.1462 | 3.0145 | 0.3130 | 0.0612 | 3.3354 | 2.1792 | 0.0573 | 3.6290 | 4.0672 | 2.3067 | 2.3002 | 3.5306 | 2.7798 | 1.6694 | 0.5520 | 0.0743 | 2.6964 | 2.5673 | 2.0518 |
|  |  |  |  |  |  |  |  |  |  |  |  |  |  |  |  |  |  |  |  |  |  |  |
| *Akkermansia* | 0.1036 | 0.0765 | 0.1710 | 0.2674 | 0.2577 | 0.1989 | 1.7002 | 0.1412 | 0.2092 | 0.1614 | 0.1440 | 0.1532 | 0.1223 | 0.0828 | 0.1315 | 0.1004 | 0.1208 | 0.2102 | 1.3925 | 0.1054 | 0.1255 | 0.1127 |
| *Bifidobacterium* | 10.8750 | 10.1012 | 16.3824 | 17.3544 | 21.5347 | 14.3152 | 12.9534 | 16.6810 | 23.7295 | 29.5023 | 15.2450 | 13.0237 | 12.7756 | 6.0913 | 13.3630 | 10.0685 | 10.5755 | 16.7589 | 14.7211 | 12.9567 | 12.9830 | 9.5149 |
| *Faecalibacterium* | 0.2164 | 0.2143 | 0.2645 | 0.3009 | 0.4520 | 0.3599 | 4.8272 | 0.2261 | 0.3457 | 0.4239 | 0.2820 | 0.3435 | 0.2214 | 0.1887 | 0.2264 | 0.2138 | 0.2731 | 0.3700 | 7.0899 | 0.2655 | 0.2544 | 0.2206 |
| *Lactobacillus* | 0.0005 | 0.0000 | 0.0000 | 0.0000 | 0.0000 | 0.0000 | 0.0065 | 0.0000 | 0.0060 | 0.0012 | 0.0000 | 0.0000 | 0.0000 | 0.0000 | 0.0000 | 0.0000 | 0.0000 | 0.0000 | 0.0218 | 0.0000 | 0.0056 | 0.0000 |

1. Shown are all phyla and all top 3 up and down regulated, as well as 4 physiologically important bacterial genera (*Akkermansia, Bifidobacterium, Faecalibacterium* and *Lactobacillus*).
2. Each vitamin was tested at 3 doses (0.2x, 1x, and 5x) (Table S2).
3. Data are shown as relative abundance. No statistical analysis was performed.
