# Supplementary material for: Effects of colon-targeted vitamins on the composition and metabolic activity of the human gut microbiome– a pilot study
Source: Gut Microbes. 2021 Feb 21;13(1):1875774. doi: 10.1080/19490976.2021.1875774 (PMC7899684; doi:10.1080/19490976.2021.1875774)
Supplement: Supplemental Material [file KGMI_A_1875774_SM7459.zip › Supplementary information/Additional file 6.docx]

**Table S6: Effect of vitamins on immunological biomarkers, including GROa-CXCL1, IL-8-CXCL8 and MIP3A-CCL20 production by HT29 cells.**

| **Cytokine** | **Vitamin** | **Dose** | **Concentration (pg/ml)** |
| --- | --- | --- | --- |
| **GROa-CXCL1** | Control | - | 250.00**^1^** |
|  | Vitamin B2 | 0.2 | -627.33 |
|  |  | 1 | 262.33 |
|  |  | 5 | 222.33 |
|  | Vitamin C | 0.2 | -455.67 |
|  |  | 1 | -184.00 |
|  |  | 5 | -211.33 |
|  | Vitamin E | 0.2 | -33.67 |
|  |  | 1 | 107.67 |
|  |  | 5 | 703.00 |
| **GROa-CXCL1** | Control | - | 170.00 |
|  | Vitamin D3 | 0.2 | 193.00 |
|  |  | 1 | 37.67 |
|  |  | 5 | 60.00 |
|  | Vitamin A | 0.2 | 86.67 |
|  |  | 1 | 41.00 |
|  |  | 5 | 123.00 |
|  | Vitamin B2+C | 0.2 | -111.33 |
|  |  | 1 | 127.33 |
|  |  | 5 | -50.90 |
|  | Folic acid | 0.2 | -130.67 |
|  |  | 1 | -64.67 |
|  |  | 5 | -172.33 |
| **IL8-CXCL8** | Control | - | 590.00 |
|  | Vitamin B2 | 0.2 | 330.00 |
|  |  | 1 | 1323.33 |
|  |  | 5 | 700.00 |
|  | Vitamin C | 0.2 | 123.33 |
|  |  | 1 | 680.00 |
|  |  | 5 | -625.67 |
|  | Vitamin E | 0.2 | 956.67 |
|  |  | 1 | 1010.00 |
|  |  | 5 | 1544.00 |
| **IL8-CXCL8** | Control | - | 1106.67 |
|  | Vitamin D3 | 0.2 | 1016.67 |
|  |  | 1 | 1040.00 |
|  |  | 5 | 782.50 |
|  | Vitamin A | 0.2 | 1366.67 |
|  |  | 1 | 1003.33 |
|  |  | 5 | 1622.00 |
|  | Vitamin B2+C | 0.2 | 893.33 |
|  |  | 1 | 1130.67 |
|  |  | 5 | -523.67 |
|  | Folic acid | 0.2 | 1306.67 |
|  |  | 1 | 676.67 |
|  |  | 5 | 1323.33 |
| **MIP3a-CCL20** | Control | - | 1406.33 |
|  | Vitamin B2 | 0.2 | 770.67 |
|  |  | 1 | 874.33 |
|  |  | 5 | 838.33 |
|  | Vitamin C | 0.2 | 517.00 |
|  |  | 1 | 684.67 |
|  |  | 5 | -193.63 |
|  | Vitamin E | 0.2 | 740.20 |
|  |  | 1 | 703.80 |
|  |  | 5 | 786.67 |
| **MIP3a-CCL20** | Control | - | 1240.00 |
|  | Vitamin D3 | 0.2 | 749.37 |
|  |  | 1 | 696.80 |
|  |  | 5 | 646.50 |
|  | Vitamin A | 0.2 | 949.27 |
|  |  | 1 | 741.77 |
|  |  | 5 | 947.33 |
|  | Vitamin B2+C | 0.2 | 1005.33 |
|  |  | 1 | 703.93 |
|  |  | 5 | -84.00 |
|  | Folic acid | 0.2 | 1092.00 |
|  |  | 1 | 1122.33 |
|  |  | 5 | 1112.67 |

1. Data are expressed as concentration (pg/ml) of GROa-CXCL1, IL-8-CXCL8 and MIP3A-CCL20. No statistic were performed.
2. Each compound was tested at 3 doses (0.2x, 1x and 5x) **(Table S2).**
