# Supplementary material for: Effects of colon-targeted vitamins on the composition and metabolic activity of the human gut microbiome– a pilot study
Source: Gut Microbes. 2021 Feb 21;13(1):1875774. doi: 10.1080/19490976.2021.1875774 (PMC7899684; doi:10.1080/19490976.2021.1875774)
Supplement: Supplemental Material [file KGMI_A_1875774_SM7459.zip › Supplementary information/Additional file 7_revised.docx]

| **Table S7. Macronutrient and micronutrient analysis at baseline** | | | | | | | | |
| --- | --- | --- | --- | --- | --- | --- | --- | --- |
|  | **Placebo**  **(n=24)** | **Vitamin A**  **(n=12)** | **Vitamin B2**  **(n=12)** | **Vitamin B2 +C**  **(n=12)** | **Vitamin C**  **(n=12)** | **Vitamin D**  **(n=12)** | **Vitamin E**  **(n=12)** | **ANOVA_P values** |
| **Macronutrients** |  |  |  |  |  |  |  |  |
| Carbohydrate Kilocal | 881.54 (65.79) | 984.55 (83.04) | 764.33 (67.52) | 844.17 (77.35) | 886.50 (79.74) | 799.36 (81.04) | 810.92 (71.60) | 0.41 |
| Carbodydrate Kilocal % | 42.73 (1.13) | 45.81 (1.21) | 42.55 (1.31) | 43.13 (1.39) | 43.47 (0.87) | 41.41 (0.83) | 39.46 (1.70) | 0.34 |
| Fat Kilocal | 801.29 (75.39) | 766.82 (87.39) | 663.75 (72.97) | 699.25 (67.32) | 761.42 (66.39) | 707.36 (58.64) | 809.33 (73.97) | 0.08 |
| Fat Kilocal % | 37.86 (0.93) | 34.93 (1.13) | 36.18 (1.54) | 35.82 (1.56) | 37.29 (0.96) | 37.42 (1.60) | 39.21 (1.21) | 0.58 |
| Protein Kilocal | 378.21 (31.11) | 391.45 (40.54) | 348.50 (33.44) | 355.08 (28.63) | 358.50 (33.07) | 359.91 (37.69) | 420.75 (45.68) | 0.79 |
| Protein Kilocal % | 18.10 (0.46) | 18.14 (0.70) | 19.32 (0.64) | 18.61 (0.95) | 17.27 (0.63) | 18.86 (1.54) | 20.35 (1.36) | 0.32 |
| Alcohol Kilocal | 24.54 (6.04) | 27.18 (10.93) | 29.17 (8.38) | 37.42 (10.94) | 35.00 (8.47) | 49.73 (15.40) | 21.08 (5.09) | 0.85 |
| Alcohol Kilocal % | 1.32 (0.35) | 1.15 (0.35) | 1.95 (0.60) | 2.39 (0.79) | 1.97 (0.58) | 2.33 (0.65) | 0.99 (0.21) | 0.33 |
| Total Kilocal | 2085.58 (164.52) | 2170.00 (208.22) | 1805.75 (158.04) | 1935.92 (154.01) | 2041.42 (171.06) | 1916.36 (170.48) | 2062.08 (167.85) | 0.70 |
| **Micronutrients** |  |  |  |  |  |  |  |  |
| Biotin (B7) µg | 51.74 (4.44) | 54.82 (4.8) | 56.6 (9.26) | 76.2 (26.56) | 49.08 (5.65) | 55.62 (4.7) | 59.28 (4.85) | 0.72 |
| Calcium mg | 1124.91 (80.79) | 1075.08 (132.47) | 1247.58 (223.64) | 1123.75 (116.45) | 1139.18 (165.8) | 1174.55 (122.32) | 1302.5 (136.87) | 0.91 |
| Carotene µg | 3701.27 (929.84) | 4507.08 (591.56) | 4380.17 (449.94) | 3751.17 (554.75) | 3871.64 (910.93) | 3497.55 (790.96) | 3795.88 (570.61) | 0.95 |
| Chloride mg | 3253.18 (457.33) | 2981.17 (280.37) | 3127 (267.99) | 2848.25 (237.29) | 2884.46 (279.1) | 3097.46 (217.6) | 3721.46 (304.14) | 0.30 |
| Copper mg | 1.24 (0.09) | 1.21 (0.11) | 1.35 (0.09) | 1.28 (0.14) | 1.17 (0.12) | 1.24 (0.09) | 1.41 (0.08) | 0.60 |
| Folates (B9) µg | 279.27 (23.4) | 272.58 (22.89) | 300.42 (28.68) | 284.08 (24.36) | 280.46 (28.54) | 288.46 (20.41) | 299.63 (16.55) | 0.97 |
| Iron mg | 11.47 (1.15) | 11.55 (1.04) | 12.3 (0.87) | 11.56 (1.26) | 10.56 (1.12) | 12.16 (0.9) | 12.8 (0.85) | 0.77 |
| Magnesium mg | 345.09 (25.31) | 347.25 (30.46) | 370 (35.16) | 402 (85.45) | 321.73 (31.92) | 359.46 (21.89) | 379.83 (23.83) | 0.87 |
| Manganese mg | 4.69 (0.53) | 4.33 (0.31) | 5.32 (0.58) | 5.18 (0.91) | 4.96 (0.71) | 4.18 (0.32) | 4.84 (0.35) | 0.78 |
| Niacin mg | 23.26 (2.27) | 23.21 (2.03) | 21.4 (1.51) | 25.74 (7.27) | 19.66 (2.36) | 24.72 (2.98) | 25.65 (1.85) | 0.82 |
| Niacin total (B3) mg | 39.36 (3.35) | 41.08 (3.42) | 39.58 (2.95) | 41.79 (8.31) | 35.99 (4.02) | 45.64 (5.64) | 45.76 (3.26) | 0.72 |
| Non-starch polysaccharides g | 17.06 (1.59) | 16.84 (1.44) | 17.08 (1.41) | 16.08 (1.77) | 15.74 (1.62) | 15.21 (1.02) | 16.23 (0.92) | 0.96 |
| Pantothenic Acid (B5) mg | 7.34 (0.53) | 6.88 (0.84) | 7.83 (1.3) | 6.47 (0.64) | 6.69 (0.81) | 7.64 (0.93) | 8.15 (0.8) | 0.76 |
| Phosphorus mg | 1591.46 (143.32) | 1565.92 (155.1) | 1689.58 (191.47) | 1561.42 (170.18) | 1501 (159.35) | 1712.82 (167.56) | 1821.54 (143.82) | 0.76 |
| Potassium mg | 4034.18 (296.03) | 3701.83 (362.71) | 3990.58 (406.8) | 4334.5 (994.91) | 3478.36 (332.97) | 3856.73 (293.33) | 4129.71 (283.17) | 0.91 |
| Retinol µg | 287.09 (34.65) | 358.58 (54.81) | 398.58 (62.14) | 518.33 (94.82) | 429.46 (69.98) | 429.64 (36.06) | 610.54 (90.79) | 0.06 |
| Riboflavin (B2) mg | 2.16 (0.19) | 2.12 (0.27) | 2.38 (0.45) | 2.16 (0.25) | 2.07 (0.23) | 2.26 (0.26) | 2.57 (0.29) | 0.86 |
| Sodium mg | 1978.55 (319.43) | 1820 (184.53) | 1879.75 (141.73) | 1724.5 (130.07) | 1793.64 (180.7) | 1908.55 (149.39) | 2306.42 (192.18) | 0.28 |
| Thiamin (B1) mg | 1.81 (0.18) | 1.6 (0.16) | 1.83 (0.16) | 1.57 (0.16) | 1.67 (0.2) | 1.65 (0.15) | 1.86 (0.13) | 0.75 |
| Tryptophan mg | 1070.82 (87.13) | 1105.25 (91.92) | 1159.83 (116.35) | 1010.08 (83.75) | 1034.18 (112.27) | 1304.18 (164.63) | 1264.46 (89.28) | 0.36 |
| Vitamin A (ret eq) µg | 1016.18 (178.42) | 1146.75 (105.97) | 1232.75 (104.25) | 1196.67 (178.38) | 1130.82 (161.85) | 1075.73 (131.98) | 1361.96 (142.24) | 0.66 |
| Vitamin B12 µg | 8.04 (0.76) | 7.97 (1.16) | 8.41 (1.56) | 7.73 (0.77) | 7.85 (1.07) | 8.5 (1.38) | 10.3 (1.15) | 0.58 |
| Vitamin B6 mg | 2.26 (0.24) | 1.95 (0.18) | 2.08 (0.18) | 1.79 (0.16) | 1.88 (0.21) | 2.06 (0.23) | 2.21 (0.16) | 0.60 |
| Vitamin C mg | 122 (19.9) | 108.94 (14.31) | 130.67 (19.35) | 108.69 (15.83) | 126.21 (21.49) | 112.55 (11.54) | 110.51 (8.38) | 0.91 |
| Vitamin D µg | 3.16 (0.49) | 4.31 (0.52) | 3.49 (0.64) | 3.47 (0.41) | 3.62 (0.55) | 3.46 (0.54) | 4.12 (0.38) | 0.66 |
| Vitamin E mg | 7.85 (0.9) | 8.54 (0.86) | 9.71 (0.84) | 7.88 (0.77) | 8.12 (0.88) | 8.89 (0.58) | 8.99 (0.58) | 0.62 |
| Vitamin K1 µg | 89.66 (15.18) | 107.58 (15.5) | 104.8 (16.81) | 93.13 (12.72) | 121.82 (28.35) | 105.68 (19.59) | 85.45 (12.74) | 0.76 |
| Zinc mg | 11.73 (1.35) | 11.26 (1.16) | 11.67 (0.96) | 10.63 (1.1) | 10.28 (1.05) | 13.46 (1.63) | 13.04 (0.99) | 0.43 |
