# Supplementary material for: Effects of colon-targeted vitamins on the composition and metabolic activity of the human gut microbiome– a pilot study
Source: Gut Microbes. 2021 Feb 21;13(1):1875774. doi: 10.1080/19490976.2021.1875774 (PMC7899684; doi:10.1080/19490976.2021.1875774)
Supplement: Supplemental Material [file KGMI_A_1875774_SM7459.zip › Supplementary information/Supplementary materials_revised.docx]

**Supplementary materials**

**Material and methods**

**Measurement of vitamin B2 concentration in plasma**

*Chemicals and reagents***:** Riboflavin (vitamin B2) was purchased from Dr. Ehrenstorfer, GmbH. Labelled internal standard ^13^C_4_ ^15^N_2_ Riboflavin was purchased from medical isotopes. Other chemicals used, were of an analytical grade and obtained from either Sigma-Aldrich or Merck Millipore. Water used was passed through a Milli-Q water purification system (Millipore).

*Instrumentation*: Separation and quantification of vitamin B2 (as riboflavin) was performed via Agilent 1290 High performance liquid chromatography (UHPLC) coupled with an API 4000 mass spectrometer (MS) from AB Sciex. A Turbo Ion Spray source operating in positive mode was used for vitamin B2 determination. Criteria for identification and quantification were retention time and multiple reaction monitoring (MRM) transitions (transition for vitamin B2 m/z 377/243; 25-OH-D3 m/z 383/211).

The analytical column used for vitamin B2 analysis was an Ascentis Express C8 from Supelco. Samples were eluted using a gradient from 100% of water to 100% of acetonitrile (both solvents were acidified with acetic acid).

*Sample preparation*: In order to remove the protein and extract the riboflavin, an aliquot of plasma sample was combined with a solution of trichloroacetic acid (50 g/L) containing the internal standard (^13^C_4_ ^15^N_2_ riboflavin). Following centrifugation, the supernatant was injected into the HPLC-MS system.

*Quantification*: Quantification was performed by applying dedicated external calibrations using internal standards for Vitamin B2. To assess the daily and long-term laboratory performance (accuracy and precision) of the method, dedicated standard and quality-control samples were analyzed daily with unknown samples.

**Measurement of vitamin B2 concentration in feces**

B2 vitamin in feces was analyzed by the Swiss Vitamin Institute, using HPLC with fluorescence detection after a solvent extraction.


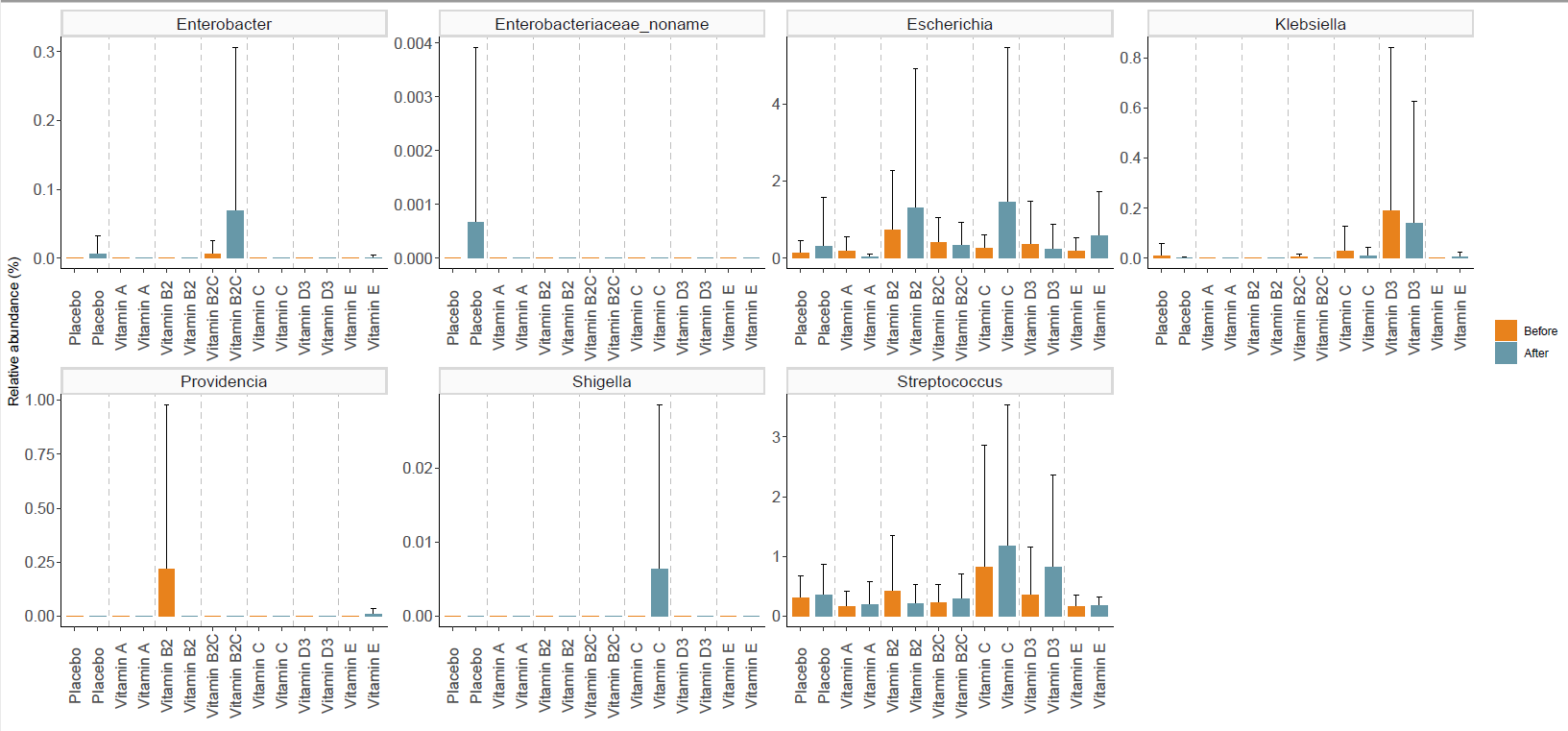


**Figure S1**: Relative abundance (%) of pathogens before and after colon-delivered vitamin intervention. Values are shown as average ± STDEV


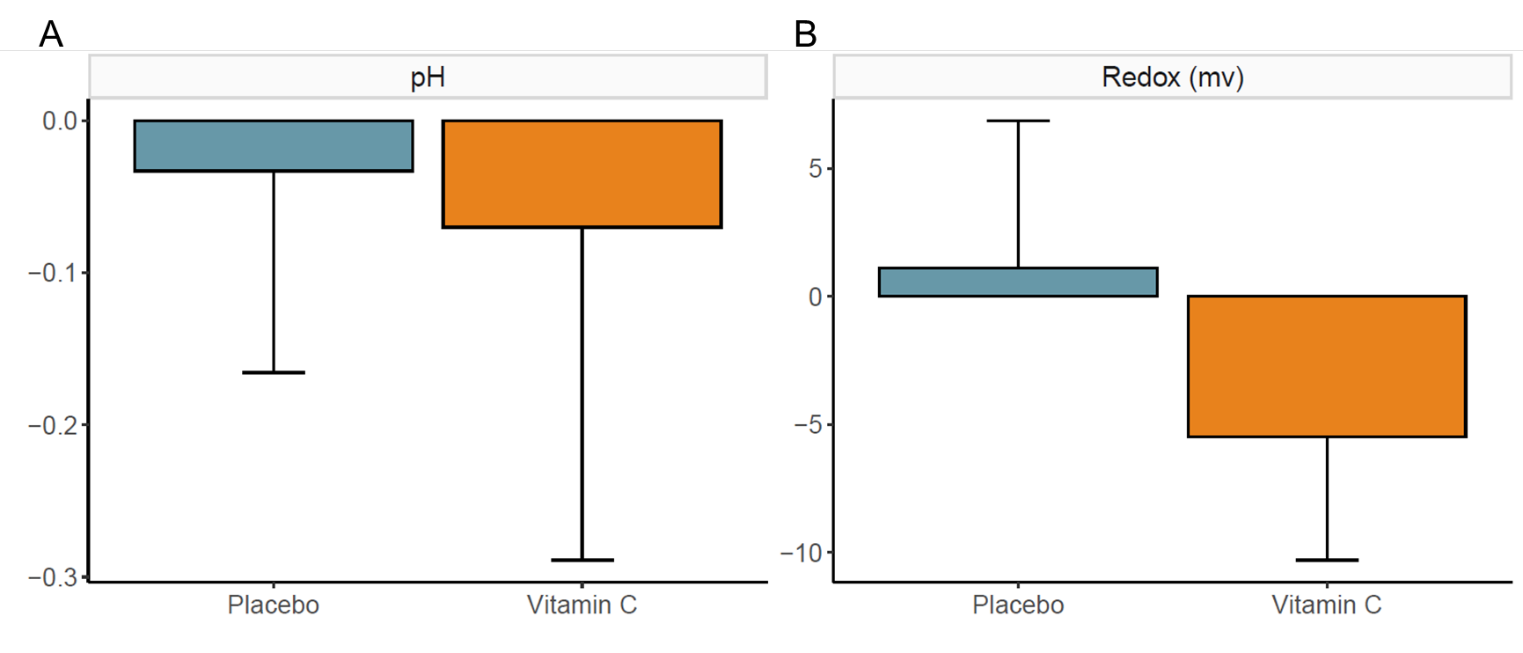


**Figure S2**: Change in pH (A) and redox potential (B) before and after vitamin C colon-delivered vitamin intervention (n=10) versus placebo (n=11). Values are shown as average ± SE.


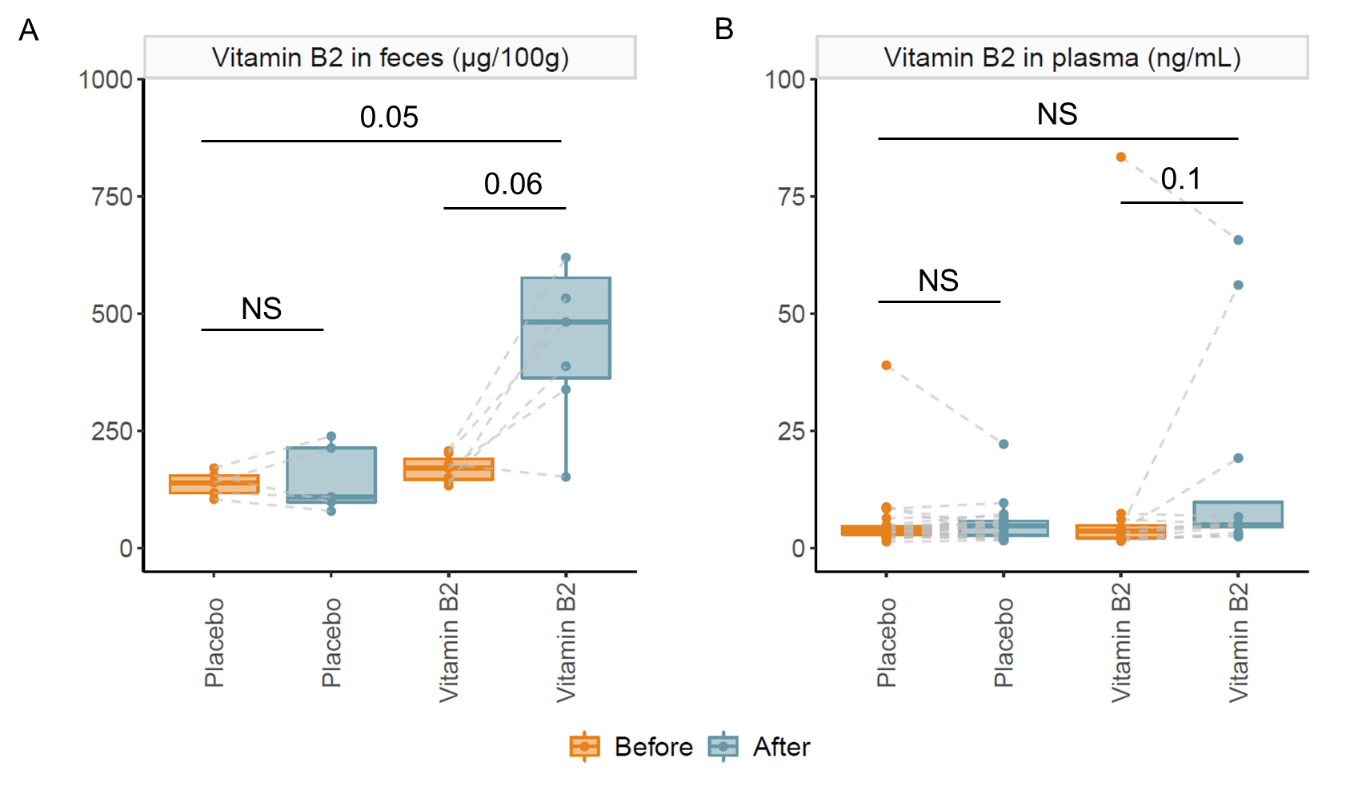


**Figure S3**: Vitamin B2 levels in feces **(A)** and plasma **(B)** before and after colon-delivered vitamin intervention. Vitamin B2 levels were compared before and after colon-delivered vitamin intervention using the paired Wilcoxon test. Absolute changes between the intervention group and the placebo using Wilcoxon test. Values are shown as median and interquartile range. NS, *p* > 0.1.

**
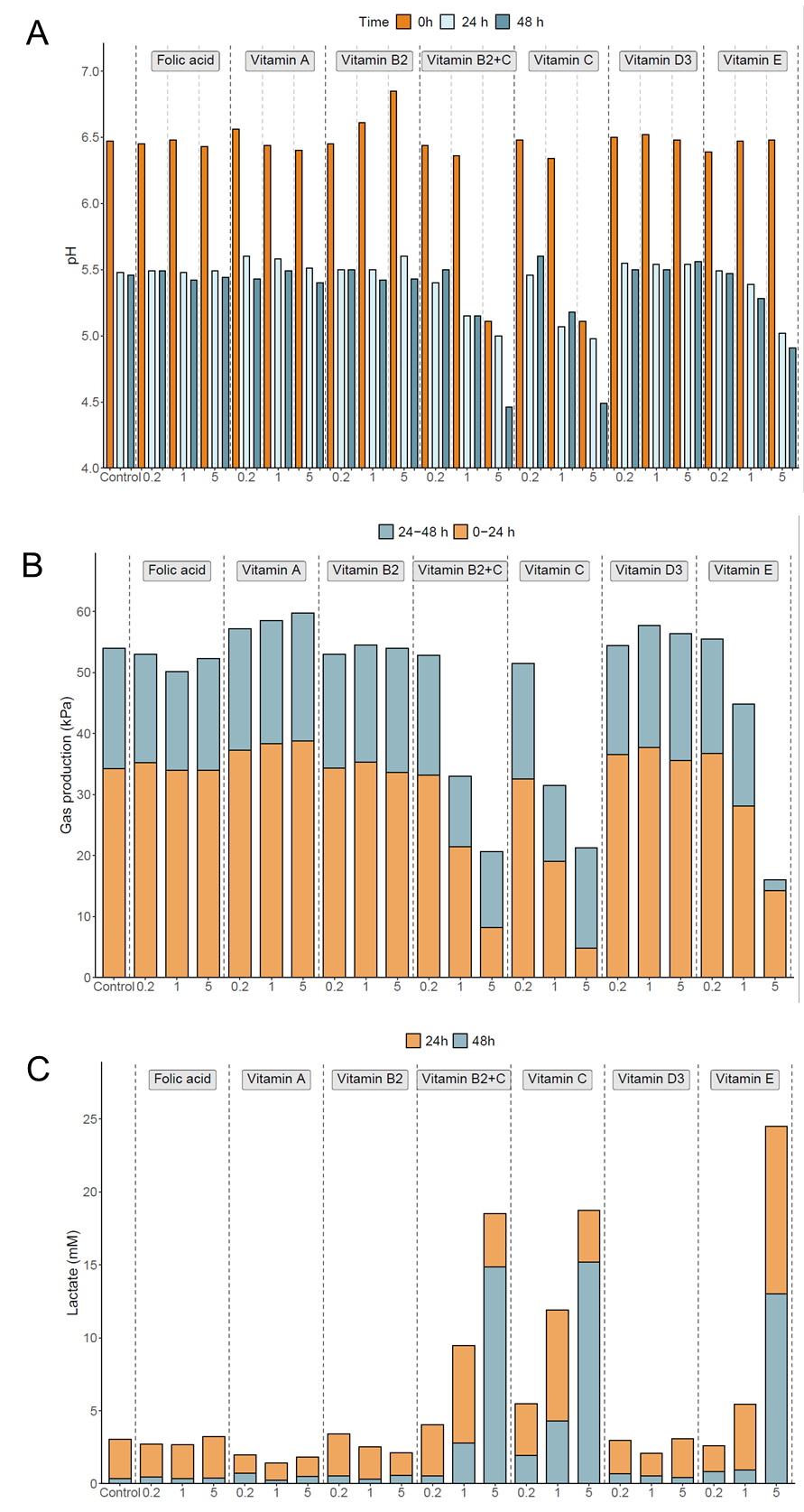
**

**Figure S4**: *In vitro* pH **(A)**, gas **(B)** and lactate production **(C)** after 48 h fermentation with the addition of vitamins. Each vitamin was tested at 3 doses (0.2x, 1x, and 5x) **(Table S2)**.
